# Amyloid-β, Tau Protein, α-Synuclein, TDP-43, and FUS in Mixed Pathology: And Intrinsic Disorder to Rule Them All

**DOI:** 10.3390/ijms27083669

**Published:** 2026-04-20

**Authors:** Alex S. Siebner, Vladimir N. Uversky

**Affiliations:** 1 Institute of Tropical Medicine, University Clinic Tübingen, 72074 Tübingen, Germany; alex.siebner@medizin.uni-tuebingen.de; 2 German Center for Infection Research (DZIF), Partner Site Tübingen, 72074 Tübingen, Germany; 3 Department of Molecular Medicine, Morsani College of Medicine, University of South Florida, Tampa, FL 33612, USA

**Keywords:** protein intrinsic disorder, neurodegeneration, proteinopathies, liquid–liquid phase separation, amyloid-β, tau, α-synuclein, TDP-43, FUS

## Abstract

Neurodegenerative diseases, including Alzheimer’s Disease (AD), Parkinson’s Disease (PD), Lewy Body Disease (LBD), and related dementias, represent a global health challenge, particularly in aging populations. The simultaneous occurrence of neurodegenerative diseases in an aging population suggests a potential link between causative proteins. Such neurodegenerative proteins, including amyloid-β (Aβ), τ-protein (tau), α-synuclein, TAR DNA-binding protein 43 (TDP-43), and Fused in Sarcoma (FUS), share key characteristics of intrinsically disordered proteins (IDPs), which can explain promiscuous physical interactions, cross-seeding, co-occurrence, pathological synergy, and shared upstream and downstream mechanisms. This review synthesizes current evidence on (1) shared biophysical features of neurodegeneration-associated proteins, (2) mechanisms driving mixed neuropathology, (3) therapeutic implications of disorder-driven interactions, and (4) key unresolved questions shaping future research. By framing neurodegeneration as a network of interacting, disorder-driven proteinopathies rather than isolated entities, this perspective highlights the need for integrative, systems-level approaches to better understand disease heterogeneity and to identify novel targets for intervention.

## 1. Introduction

### 1.1. Clinical and Pathological Motivation

Neurodegenerative diseases are a diverse group of maladies, varying by pathology, clinical symptoms, and genetics. Until now, they have lacked effective disease-modifying treatments. Mechanistically, they are classified as proteinopathies characterized by the accumulation of specific, misfolded proteins that aggregate into toxic, ordered structures in glial cells, neurons, or extracellularly. Some of these diseases share similar pathologies, where the same misfolded protein deposits in different brain regions and causes distinct cognitive and/or motor neuronal impairments, as illustrated by so-called synucleinopathies, e.g., Parkinson’s Disease (PD), Dementia with Lewy Bodies (DLB), Multiple System Atrophy (MSA), Pure Autonomic Failure (PAF), and Rapid Eye Movement (REM) Sleep Behavior Disorder (RBD) [1], as well as tauopathies, such as Alzheimer’s disease (AD), frontotemporal dementia (FTD), Pick’s disease (PiD), chronic traumatic encephalopathy (CTE), Progressive Supranuclear Palsy (PSP), Argyrophilic Grain Disease (AGD), and corticobasal degeneration (CBD) [2]. Different pathologies are associated with the accumulation of aggregated forms of α-synuclein and τ-protein (tau) in different brain regions [3]. However, it is increasingly recognized that neurodegenerative diseases often involve multiple co-occurring proteinopathies rather than a single, isolated one [4]. In fact, around 75% of autopsies revealed multiple neuropathologies in older adults, highlighting a pressing global health concern in a worldwide-growing aging population [5]. This mixed (neuro)pathology seems to be the rule rather than the exception [6] and makes diagnosis and treatment difficult, e.g., for AD, PD, Lewy Body Disease (LBD), Vascular Brain injury (VBI), or dementia. Since such mixed pathology is most seen in dementia, it is frequently referred to as “mixed” dementia. This means the simultaneous occurrence of several distinct brain diseases in one patient, which suggests that proteinopathies do not occur in isolation or independently but rather with other diseases [7,8]. In these cases, hallmark markers of AD (amyloid plaques and tau tangles) often overlap with other issues, such as vascular damage (blood vessel disease) or the accumulation of additional proteins, α-synuclein (Lewy bodies), TAR DNA-binding protein 43 (TDP-43), and Fused in Sarcoma (FUS) [4]. The synergistic effect of multiple pathologies commonly results in more rapid, severe dementia, underlining why many cases involve complex, multifactorial, or “mixed” dementia rather than pure AD. Since the prevalence of mixed pathologies increases in the context of an aging population, and since the related pathologies are associated with the misbehavior of specific proteins, these findings need to be better understood, particularly considering the phenomenon of protein intrinsic disorder (PID), which can shed additional light on neuropathologies.

PID refers to the property of many proteins or protein regions to not adopt a single, stable three-dimensional (3D) structure under physiological conditions but still be fully functional, with intrinsically disordered proteins (IDPs) and intrinsically disordered regions (IDRs) being the categories within this framework [9,10,11,12,13,14,15,16,17,18,19,20,21,22,23,24,25,26]. IDPs are proteins that entirely lack a stable fold, while IDRs are segments within otherwise structured proteins that exhibit disorder. Key characteristics of IDPs are (1) structural features, e.g., high conformational flexibility, interconverting conformations, lack of a stable folded 3D structure, and sensitivity to environmental conditions, such as pH, ions, and crowding [10,11,12,14,15,27,28]; and (2) sequence peculiarities, e.g., high content of charged and polar amino acids (glutamic acid (E), aspartic acid (D), lysine (K), arginine (R), glutamine (Q), serine (S)), and therefore low hydrophobic content and depletion in bulky hydrophobic residues (isoleucine (I), leucine (L), valine (V), phenylalanine (F), tryptophan (W)), low sequence complexity, and the presence of repetitive motifs [11,29,30,31,32]. Therefore, the absence of regular structure in these proteins has been explained by these specific features of their amino acid sequences, including the presence of numerous uncompensated charged groups (often negative), i.e., a high net charge at neutral pH, arising from the extreme pI values in such proteins [33,34,35], and a low content of hydrophobic amino acid residues [30].

IDPs/IDRs are known as promiscuous binders capable of interaction with a variety of binding partners, including other proteins, membranes, nucleic acids, and various small molecules, and as a result, they have multiple biological functions [36]. Many IDPs/IDRs are capable of undergoing at least partial disorder-to-order transition upon binding [37], whereas others retain a considerable level of disorder even in their bound states, forming “fuzzy” complexes [38,39,40,41,42,43,44,45,46,47]. IDPs/IDRs often act as hub proteins with many connections and form complex protein–protein interaction (PPI) networks [48,49,50,51,52]. Among the important biological functions of IDPs/IDRs, which are complementary to the activities of ordered proteins and domains [9,11,13,17,53], are the regulation of cell division, transcription and translation, signal transduction, the storage of small molecules, and chaperone activity [54,55,56,57,58,59,60,61]. These proteins regulate the function of binding partners and promote the assembly of various complexes [62,63,64], ranging from the BAF complex [65], mediator complex [66], and mitochondrial enzymatic machines [67] to spliceosomes [68], nucleosomes, histones [69], and ribosomes [70]. Biological functions of IDPs/IDRs are fine-tuned by various post-translational modifications (PTMs) [71,72,73].

Recent studies indicated that IDPs/IDRs serve as fundamental drivers of liquid–liquid phase separation (LLPS). LLPS is a physical, highly dynamic, controllable, and reversible phenomenon that plays a fundamental role in a wide spectrum of biological processes. LLPS is linked to the biogenesis of various membrane-less organelles (MLOs), which are also known as biomolecular condensates (BCs), granules, intracellular microdomains, speckles, bodies, puncta, coacervates, and naked cellular organelles, among other names. Such biomolecular condensates represent a distinct dense phase that coexists with a surrounding dilute phase, driven by the thermodynamic imperative to minimize the system’s free energy. These dynamic, membrane-less compartments within cells facilitate spatial organization by separating specific proteins and nucleic acids from the surrounding cytoplasm or nucleoplasm [74,75,76,77,78,79,80,81,82,83,84,85,86]. Because they lack a surrounding lipid membrane, their molecular constituents (proteins and RNA) can readily exchange with the surrounding “dilute phase” on a timescale of seconds to minutes [87,88,89,90,91,92,93,94]. They are rapidly formed and dissolved via LLPS, enabling cells to respond quickly to stimuli, a feature vital for the innate immune system [95,96], cancer progression [97,98], and health and disease in general [74]. LLPS is orchestrated by transient, weak, multivalent interactions among biological macromolecules, particularly proteins with IDRs, RNA, and DNA. These interactions include electrostatic forces, cation–π interactions, and hydrophobic effects [74]. By rapidly concentrating constituents within these liquid-like droplets, phase separation enhances reaction kinetics and organizes signaling networks (e.g., in immune response and transcriptional regulation) [99]. Cells control phase separation through PTMs (e.g., phosphorylation), changes in the local concentration of constituents, and molecular chaperones that act as “dissolvases” [100,101,102].

Often, LLPS is activated when cells experience stress, and thereby it represents a protective mechanism [86,103]. The droplet-like structures formed as a result of LLPS limit the interaction volume of the molecules and increase the probability of interaction [86]. Many MLOs are expected to be present in a certain location at a certain time [86]. Beyond merely operating within favorable conditions, MLOs are characterized by a distinct timeframe and specific requirements for secure existence, alongside the “comfort zone” of the conditions favorable to LLPS [86]. When MLOs persist beyond their intended functional lifespan, they can undergo a “pathological aging” process. This transformation may trigger neurodegenerative diseases by turning these organelles into hubs for the accumulation of toxic amyloidogenic proteins [86]. Other triggers for pathological LLPS and abnormal MLOs are increased levels of proteins that undergo LLPS, irregular PTMs, specific disease-linked mutations, or chromosomal translocations [86]. A key finding in the field is the prevalent association of intrinsic disorder with disease. It ranges from cancer and infectious diseases to cardiovascular disease and neurodegeneration [104,105,106,107,108].

### 1.2. Purpose and Scope of the Review

**Figure 1:**
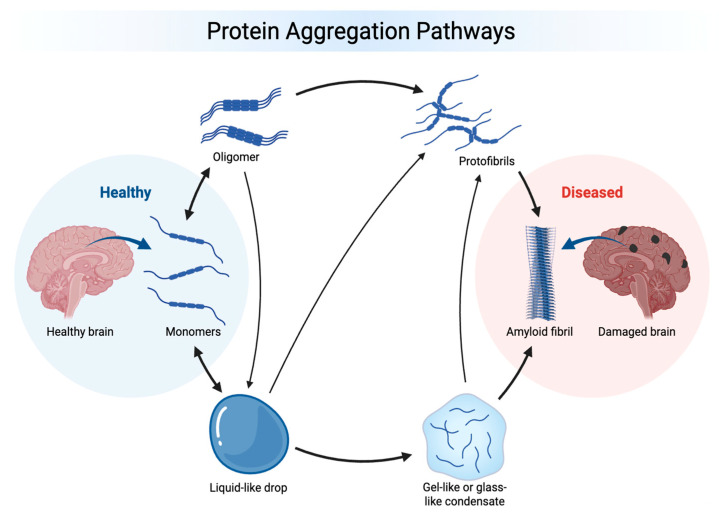
Proteins in neurodegeneration exist on a continuum, where normal, soluble proteins gradually misfold into insoluble, pathogenic aggregates that trigger disease, marking a transition from healthy cellular homeostasis to neurodegeneration. The processes of misfolding and aggregation are sometimes reversible. Proteins can undergo LLPS, leading to the formation of dynamic, liquid-like condensates. Sometimes, these assemblies can age/mature from liquid-like droplets into more gel-like or glass-like condensates. Those forms are intermediates for protofibrils and insoluble amyloid fibrils. Cellular dysfunction and neurodegenerative diseases are caused by misfolding, aggregation, and amyloid fibril formation, as evidenced by the transition from a healthy to a diseased brain state. Arrows through the different states indicate reversible and irreversible transitions between aggregation states. Created with BioRender.com (accessed on 22 January 2026).

This review provides a comprehensive evaluation of the five major neurodegeneration-related, aggregating proteins involved in mixed pathology, such as amyloid beta (Aβ), tau, α-synuclein, TDP-43, and FUS, within the context of PID. Figure 1 illustrates an accepted model linking misbehavior and aggregation of the neurodegeneration-related proteins with the transition from a healthy to a diseased brain state. Misbehavior of these proteins that can occur individually or in combination can be better understood by applying the PID concept, which aids in explaining the underlying regulatory failures. This raises questions about physical interactions, cross-seeding, co-occurrence, pathological synergy, and shared upstream and downstream mechanisms with a unique view on PID and its neuropathological impact. Here, we will discuss those proteins in the light of functional advantages and disadvantages such as (1) binding promiscuity: IDPs can interact with many different partners and one region can bind multiple targets using different conformations; (2) molecular recognition flexibility, where binding often occurs via disorder-to-order transitions and enables context-dependent interactions; (3) regulatory versatility as ideal substrates for various PTMs, e.g., phosphorylation, acetylation, ubiquitination, and SUMOylation; and (4) multifunctionality, in which the same protein participates in transcription, RNA metabolism, signaling, and stress responses, forming stress granules. All of these interactions need to be considered when referring to neuropathological proteins. In the past, the population prevalence of the co-occurrence of the hallmarks of different proteinopathies was reported in mixed pathologies [4,109,110], which seems to not be sufficient. We introduce a selectivity model, contributing to a mechanistic understanding.

## 2. Overview of the Five Neurodegeneration-Associated Proteins and Their Intrinsic Disorder Status

### 2.1. Amyloid-β (Aβ)

Amyloid-β (Aβ) is a peptide abundantly produced in the brain, with the Blood–Brain Barrier (BBB) serving as a critical gateway that regulates the bidirectional movement (influx and efflux) of existing Aβ peptides between the brain and the peripheral blood circulation [111,112,113]. It is derived from the type I transmembrane amyloid precursor protein (APP) via the sequential cleavage by β-secretase (BACE1) and γ-secretases. Low concentrations of soluble forms of Aβ contribute to the normal neuronal activity [114]. Various forms, such as Aβ_40_ (40-residue-long peptide that corresponds to the residues 672–711 of the APP), Aβ_42_ (42-residue-long peptide corresponding to the APP residues 671–712), N-truncated Aβ_4–42_, and amyloid-α (also known as Aβ_17–40/42_ or p3) and different oligomeric and aggregated states, such as non-fibrillar or soluble forms, amyloid fibrils, and amorphous aggregates are known. Among these, Aβ_42_ aggregates more readily, whereas oligomeric Aβ species are particularly associated with neurotoxicity and the development of AD and related neuropathologies [115].

Aβ is an intrinsically disordered peptide as a monomer, whose amino acid sequence is characterized by a charged, flexible N-terminus and a hydrophobic C-terminal region containing aggregation-prone motifs [116]. This sequence organization underlies its conformational plasticity, binding promiscuity, and strong tendency toward β-sheet-rich self-assembly [117]. The presence of additional hydrophobic residues in Aβ_42_ further enhances aggregation propensity, providing a molecular explanation for its increased pathogenicity [118]. Furthermore, PTMs, such as phosphorylation and acetylation, can significantly influence the aggregation of Aβ. It has been shown that PTMs could modulate the polymerization rates of Aβ and thus impact its neurotoxicity. Thus, the peculiarities of the amino acid sequence of Aβ directly link PID with aggregation and neurotoxicity [119]. Aβ aggregation emerges from the plasticity of a disordered peptide, not from the destabilization of a folded state [120,121,122]. It was also indicated that clinical variability of AD, at least in part, can be associated with the possibility of misfolded Aβ to acquire different conformations (referred to as “Aβ strains”) [123]. High levels of aggregated Aβ are associated with cognitive decline, dementia, and AD, and misfolded Aβ species can promote the misfolding of other aggregation-prone proteins, particularly tau, thereby accelerating disease progression through prion-like mechanisms [124,125,126,127,128].

AD is characterized by the accumulation of extracellular Aβ plaques and intracellular tau-containing neurofibrillary tangles (NFTs). Both contribute to impaired neuronal communication and synaptic function. The imbalance in proteostasis between Aβ production and clearance leads to the accumulation of extracellular Aβ, while abnormal tau phosphorylation leads to intracellular NFT formation, showing synergistic effects [129,130].

The pathology of AD rarely occurs in isolation, especially in aging populations. Neuropathological and biomarker-based studies indicate that Aβ plaques and tau NFTs frequently coexist with additional proteinopathies, including α-synuclein-positive Lewy pathology and TDP-43 inclusions. Moreover, increasing evidence implicates RNA-binding proteins, such as TDP-43 and FUS, in overlapping neurodegenerative processes. It links classical amyloid and tau pathology to dysregulated RNA metabolism and stress-granule dynamics. Biomolecules, such as Aβ, tau, α-synuclein, TDP-43, and FUS, can interact and exhibit prion-like seeding properties, cross-aggregation, and shared proteostatic pathways, thereby amplifying cellular dysfunction [3]. These convergent mechanisms support the view that AD is not driven by a single misfolded protein but rather arises from a broader network of interacting IDPs [131]. Those connections will be elucidated further below.

**Figure 2:**
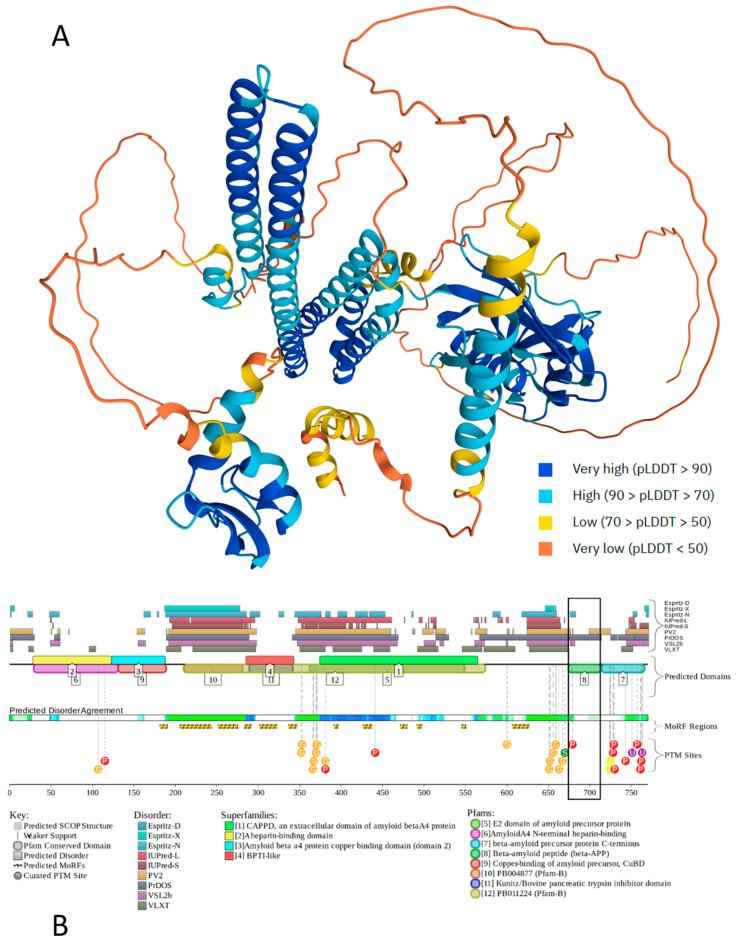
Intrinsic disorder status of human amyloid precursor protein (APP) (UniProt ID: P05067). (**A**) 3D structural model generated for human APP by AlphaFold [133]. The structure is colored based on the per-residue model confidence (p_LDDT_), which ranges between 0 and 100. Regions with very high (p_LDDT_ > 90), high (90 > p_LDDT_ > 70), low (70 > p_LDDT_ > 50), and very low model confidence (p_LDDT_ < 50) are shown by blue, cyan, yellow, and orange colors, respectively. Regions with low p_LDDT_ may be unstructured in isolation. (**B**) Functional disorder profile generated by D^2^P^2^ [134], showing the outputs of several disorder predictors such as PONDR^®^ VLXT, PONDR^®^ VSL2b, PrDOS, IU-Pred and Espritz. Consensus disorder predictions are shown by the blue–green–white bar, where blue indicates regions where the disorder predictions intersect the SCOP domain prediction and green indicates regions representing a disorder that is not found within a predicted SCOP domain. The colored bar highlighted by blue and green shades represents the consensus disorder prediction. Above this consensus bar, lines with numbered, colored bars show the predicted locations of SCOP (Structural Classifications of Proteins) domains. Yellow zigzagged bars show positions of MoRFs, whereas colored circles at the bottom of the plot show the positions of predicted PTMs. The position of Aβ, which is located within the C-terminal part of the protein, is shown within the black box.

Figure 2 illustrates the intrinsic disorder status of human amyloid precursor protein (APP). This protein operates as a cell surface receptor on neurons, facilitating critical physiological processes such as neurite outgrowth, neuronal adhesion, and axonogenesis [132]. Furthermore, transcellular interaction between APP molecules on adjacent cells can promote synaptogenesis [132].

Figure 2 shows that APP is predicted to have a high level of intrinsic disorder. This is evidenced by the presence of long regions with low and very low confidence scores (p_LDDT_ (Predicted Local Distance Difference Test) below 70) (see Figure 2A) and by the multiple IDRs confidently predicted (see Figure 2B). IDRs are very prominent, especially in the central part of APP, which is also predicted to contain 12 disorder-based PPI sites (molecular recognition features (MoRFs), which are disordered regions undergoing folding at binding to specific partners) and multiple different PTMs, indicating that intrinsic disorder is utilized by APP for its binding functions, which are regulated by PTMs. Likely, because of these features, APP is capable of interaction with a very broad spectrum of proteins (e.g., according to BioGRID [135,136], it has more than 2400 protein partners). Although Aβ peptides are lipophilic, they are also predicted to contain some disorder, which might be utilized in coordination of Cu^2+^ and Zn^2+^ ions by these metal chelators with metal-reducing activity [137]. As per the FuzDrop analysis [138], APP is expected to spontaneously undergo LLPS (i.e., being characterized by a probability of spontaneous LLPS, p_LLPS_, of 0.7463, it can act as a droplet driver) and contain five droplet-promoting regions (DPRs, residues 188–216, 230–285, 353–373, 437–451, and 624–657), indicating that LLPS is included in its functional repertoire. Curiously, although most disease-causal mutations of APP occur within the Aβ-coding region or in its immediate proximity, it was recently shown that mutations in the N-terminus of APP protein might have pathological consequences as well, as they can promote AD-like tau pathology and notably alter the LLPS of intracellular tau [139].

### 2.2. τ-Protein (Tau)

τ-Protein (Tau) is a microtubule-associated protein that stabilizes the structure and regulates the function of microtubules. However, recent research indicated that Tau does not merely anchor microtubules in axons. Instead, it protects dynamic, labile regions from excessive stabilization by other proteins, acting as a regulator that keeps microtubule tracks flexible to ensure healthy neuronal transport [140]. This protein does much more than interact with microtubules. It acts as a signaling hub, a scaffold protein, a regulator of motor proteins (like kinesin and dynein), and even plays roles in DNA/RNA protection within the nucleus [140]. The understanding of the sequence feature and structure of tau can provide valuable insights into the biological functions and pathology caused by this protein. It has a high conformational flexibility because of the presence of charged and polar amino acids, such as aspartic acid (D), glutamic acid (E), lysine (K), serine (S), and arginine (R), while being low in hydrophobic amino acids like leucin (L), isoleucine (I), phenylalanine (F), valine (V), tryptophan (W), and tyrosine (Y). These amino acid biases prevent the formation of stable 3D structures, highlighting tau as an IDP that maintains the dynamic flexibility required for its cellular functions.

Tau is classified as an IDP that lacks a stable three-dimensional structure in its native, monomeric form, existing instead as a dynamic ensemble of interconverting conformers. This inherent structural flexibility allows it to contain IDRs that can adopt multiple structural states [28,36,141], facilitating binding to numerous partner proteins. This shows how this IDP functions as an interaction hub in cellular networks [36,141,142,143]. Tau’s plasticity leads to engagement in diverse interactions and connections with microtubules and other cellular proteins. This depends on environmental factors, such as pH, ionic strength, and macromolecular crowding, which complicates its behavior in cells, their environment and with other proteins contributing to mixed proteinopathies [144].

Tau undergoes LLPS driven by multivalent electrostatic interactions, leading to the formation of membrane-less liquid-like droplets. Phase-separated Tau can transit into β-sheet-rich fibrillar aggregates because of its repetitive motifs, which leads to more condensed amyloid structures under certain conditions, e.g., cellular stress [145,146,147,148,149,150,151,152,153,154,155,156,157,158]. Positively charged amino acids of tau contribute to LLPS, remaining essential for both cell signaling and pathology [86]. PTMs, especially phosphorylation, are key drivers to expand tau’s interaction properties and structural behavior [28,36,37,159]. Acetylation of tau is a driver of neurodegenerative disease [160]. In neurodegenerative diseases such as AD and tauopathies, it undergoes hyperphosphorylation, loses its ability to bind to microtubules, and aggregates into NFTs that consist of paired helical filaments (PHFs) [161]. This leads to aggregation, the formation of NFTs, and prion-like spreading [162,163]. For example, the abnormal accumulation of NFTs after chronic traumatic encephalopathy (CTE) disrupts normal cellular function. As mixed pathology concepts suggest, Aβ initiates a pathophysiological change leading to tau aggregation. Interestingly, NFTs of the brain’s neocortex are more related to cognitive decline than amyloid plaques [163], and Aβ plaques influence tau pathology in a mixed-pathology manner by facilitating tau aggregation processes in the presence of misfolded tau seeds [164].

In summary, low sequence complexity, repetitive motifs, and tau’s amino acids contribute to its flexibility and functional diversity and enable tau to participate in various cellular processes, including the organization of cytoskeletal formation [165]. In the context of neurodegenerative diseases like AD or tauopathies, the IDRs of tau are particularly important, as they can aggregate into neurotoxic fibrils, highlighting the dual nature of PIDs [17].

**Figure 3:**
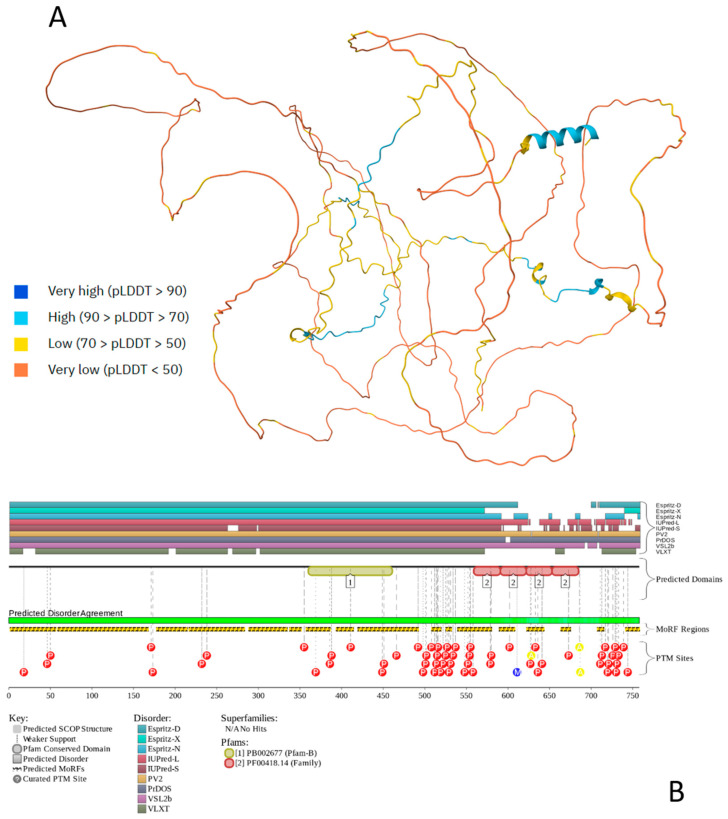
Evaluation of intrinsic disorder in human microtubule-associated protein tau (UniProt ID: P10636). (**A**) 3D structural model generated for human tau by AlphaFold. (**B**) Functional disorder profile generated by D^2^P^2^.

Figure 3 provides an outlook on the prevalence of functional disorder in human tau protein and shows that this protein is mostly disordered and contains multiple PTMs. Furthermore, according to Figure 3B, tau’s almost entire sequence is expected to be involved in disorder-based interactions and therefore can serve as a disordered scaffold. In line with these predictions, BioGRID [135,136] indicates that tau has more than 1100 protein partners. According to FuzDrop, human tau protein is clearly defined as a strong droplet driver, since it is predicted to have a very high p_LLPS_ of 0.9985 and contain four DPRs (residues 1–30, 309–589, 608–622, and 719–739) that cover almost the entire sequence of this protein.

### 2.3. α-Synuclein

α-Synuclein is a 140-amino-acid-long protein that was originally identified as a non-Aβ component (NAC) precursor (NACP). It is predominantly found in presynaptic terminals in the brain and neuronal cell bodies, where it is involved in synaptic vesicle transmission and vascular regulation. α-Synuclein is involved in transport processes in synapses and exists as multiple proteoforms, generated by truncation, PTMs, and aggregation states. These proteoforms influence aggregation and toxicity and are an ideal example of an IDP [166]. Under physiological conditions within neurons, α-synuclein is natively unfolded, lacking a stable 3D conformation, which allows aggregation to amyloids and connection to lipid membranes [35,167,168,169,170].

The primary sequence of α-synuclein is enriched in polar and charged amino acids, particularly in its N-terminal region, with a high density of lysine (K), serine (S), and glutamic acid (E) residues. This amino acid composition contributes to its low hydrophobicity and helps maintain a flexible structure without stable α-helices or β-sheets [171].

The protein contains three functional regions, such as an amphipathic N-terminal region (residues 1–60) containing an 11-residue repeat including the KTKEGV motif. Another region allows the protein to bind to acidic lipid membranes and forms α-helices (residues 61–95) and so-called NAC, a highly hydrophobic, aggregation-prone region. Its highly acidic and proline-rich C-terminal region (residues 96–140) is involved in regulating solubility, interacting with metal ions [171], and binding to protein partners in an environment-dependent manner [166,167]. Furthermore, the NAC region of α-synuclein has repetitive motifs, which promote, on the one hand, its flexible interaction with lipid membranes and other cellular components, and on the other hand, its aggregation to amyloid fibrils and its pathogenic forms [144,172], enabling it to engage in multiple PPIs crucial for its function within the brain [173]. The adoption of multiple conformations can stabilize intermolecular interactions [144].

This “chameleon” protein [167] is highly conformationally flexible and characterized by its existence in various structural conformations [168]. The protein has a strong aggregation potential and its ability to form amyloid fibrils is enhanced through different factors. Its intrinsic disorder allows the protein to adopt different conformations depending on intrinsic (negatively charged membranes, metal ions) and extrinsic environmental factors (herbicides and pesticides), such as the presence of negatively charged membranes [171,174,175,176]. Now, they undergo a change to an α-helical structure [177,178,179].

α-Synuclein can undergo LLPS [180,181,182,183,184]. Phase-separated droplets can convert into oligomers and amyloid fibrils, known for their implications in neurodegenerative diseases. Oligomers disrupt neuronal function and are toxic in neurons in vivo [185,186]. Interestingly, the generated pre-fibrillar oligomers are often more toxic than mature fibrils, emphasizing the pathological implications of transient conformational states [186].

PTMs, such as N-terminal acetylation, have been shown to influence the aggregation of α-synuclein dynamics [187]. Additionally, the interactions with chaperones can alter the aggregation propensity of α-synuclein [144,169].

PD and other synucleinopathies, such as LBD and dementia with Lewy bodies, are based on *SNCA* gene mutations. The decline in the clearance capacity of the ubiquitin–proteasome and the autophagy–lysosomal systems, together with mitochondrial dysfunction, has been indicated as a major pathophysiological mechanism of PD neurodegeneration [188]. In mice, misfolded α-synuclein acts in a prion-like manner and induces the misfolding of proteins in neighboring cells [189]. The interaction of distinct α-synuclein strains and tau impacts neurodegeneration [190]. Other studies show the formation of heterotypic droplets composed of α-synuclein and tau, and at physiologically relevant mole ratios that mimic neurons’ soma and terminal buttons, which means that heterotypic LLPS of tau and α-synuclein can be implicated in overlapping neuropathologies, which contribute to mixed pathologies [191]. In cerebrospinal fluid (CSF), α-synuclein functions as a biomarker for cognitive decline, and total tau/α-synuclein and phosphorylated ratios of tau/α-synuclein can contribute to the discrimination of PD [192].

**Figure 4:**
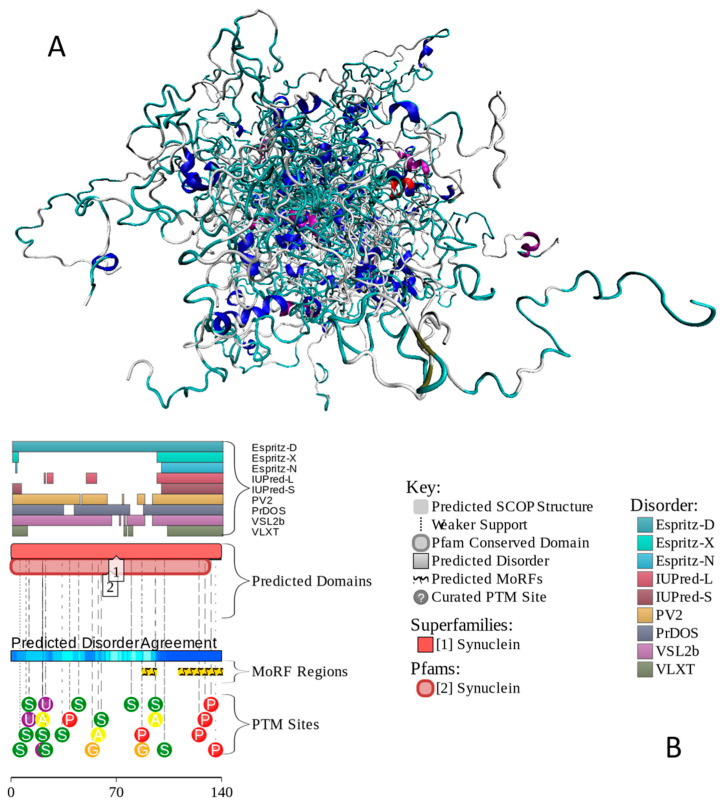
Evaluation of the intrinsic disorder predisposition of human α-synuclein (UniProt ID: P37840). (**A**) 3D conformational ensemble generated by AFflecto (https://moma.laas.fr/applications/AFflecto/, accessed on 22 January 2026) [193]. By analyzing the structural properties of the AlphaFold model, AFflecto identifies IDRs based on the p_LDDT_ score (i.e., based on the analysis of structural context) and classifies them as tails, linkers, or loops. To explore the conformational diversity of these flexible regions, AFflecto employs computationally efficient stochastic sampling algorithms [193]. It also incorporates a method to identify conditionally folded IDRs that AF may incorrectly predict as natively folded elements [193]. Therefore, AFflecto generates protein ensembles that provide a realistic representation of protein structural heterogeneity [193]. The ensemble includes 28 models. The plot was generated using Visual Molecular Dynamics (VMD) software (Version 1.8.7) for molecular visualization [194]. (**B**) Functional disorder profile generated by D^2^P^2^.

A deeper comprehension of α-synuclein’s structure, sequence, conformational flexibility, propensity to undergo LLPS, and aggregation capacity provides a framework for targeted therapeutic strategies aimed at mitigating α-synuclein-associated neurodegenerative diseases. Different roles of intrinsic disorder in multifunctionality and polypathogenicity were discussed in a comprehensive review, where it was emphasized that the remarkable structural, functional, and dysfunctional multifaceted nature of this protein can be understood using the intrinsic disorder-based proteoform concept [166]. Figure 4 illustrates these points by showing a conformational ensemble generated for human α-synuclein by AFflecto (Figure 4A) and a functional disorder profile generated by D^2^P^2^ (Figure 4B). High binding promiscuity of α-synuclein is illustrated by the fact that, according to BioGRID, it is involved in interactions with more than 1500 protein partners. With the p_LLPS_ of 0.6249 and a long IDR (residues 101–140), human α-synuclein is expected to serve as a droplet driver capable of spontaneous LLPS.

### 2.4. TAR DNA-Binding Protein 43 (TDP-43)

Transactive response (TAR) DNA-binding protein 43 (TDP-43) is a ubiquitously expressed, highly conserved, 414-amino-acid-long RNA- and DNA-binding protein, important for alternative splicing, mRNA stability, transport processes, and translation. It has a central role in neuronal RNA homeostasis and stabilizing stress granules under physiological conditions. The protein exists in a range of conformations, facilitating its involvement in the regulation of gene expression and RNA processing [7,195,196].

The C-terminal low complexity region has a high content of charged and polar amino acid residues, such as glycine (G), glutamine (Q), and asparagine (N), contributing to its low hydrophobicity [197], typically for IDPs [141]. TDP-43 contains two RNA recognition motifs (RRMs), critical for its binding to RNA/DNA and exerting its role on mRNA, as well as forming ribonucleotide granules [197]. The C-terminal domain is also called the Low-Complexity Domain (LCD) prion-like domain (PrLD), promoting PPI with other factors as well as FUS [197]. Under pathological conditions, it can be sequestered into the cytoplasm and cleaved into C-terminal fragments, which are abnormally hyperphosphorylated and subsequently aggregate, forming intracellular inclusions [198]. The sequence of TDP-43 includes IDRs, showing the “two faces” of intrinsic disorder in physiology and pathology that allow significant conformational flexibility [78,199,200]. A deeper understanding of TDP-43’s intrinsic disorder can aid in developing new therapeutic approaches for TDP-43 proteinopathies [196]. The co-occurrence of proteinopathies was already highlighted but remains to be better understood [201,202].

TDP-43 can undergo LLPS, which leads to the formation of MLOs [78,103,197,203,204,205,206,207,208,209,210,211,212,213,214,215,216]. Over time, these dynamic forms can solidify into abnormal aggregates, which is a hallmark of neurodegenerative diseases.

PTMs play a critical role in neurodegenerative disease. Aggregated TDP-43 in ALS, FTD, and AD is hyperphosphorylated in its C-terminal IDR, while physiological TDP-43 is phosphorylated at a lower level [217]. TDP-43 misfolding is connected to the translocation of the protein from the nucleus to the cytoplasm due to stress, as has been seen for tau [218]. Evidence suggests that TDP-43 occurs alongside Aβ, tau, and α-synuclein in older individuals and has been shown to contribute to mixed pathologies within the aging brain [219]. TDP-43 pathology is a defining feature of ALS and FTLD, but it is also highly prevalent in aging-related neuropathologies, and mutations in the *TARDBP* gene, coding for TDP-43, were found in patients with FTLD [220]. Autopsy studies indicate that TDP-43 inclusions are present in approximately 40–50% of AD cases and in 20–30% of cognitively normal individuals over 80 years [221]. This age-associated presentation, termed limbic-predominant age-related TDP-43 encephalopathy (LATE) [222], frequently coexists with Aβ and tau pathology and is associated with disproportionate hippocampal atrophy and accelerated cognitive decline. These epidemiological findings identify TDP-43 as one of the most common contributors to mixed neuropathologies and proteinopathies in late-life dementia [198,223].

**Figure 5:**
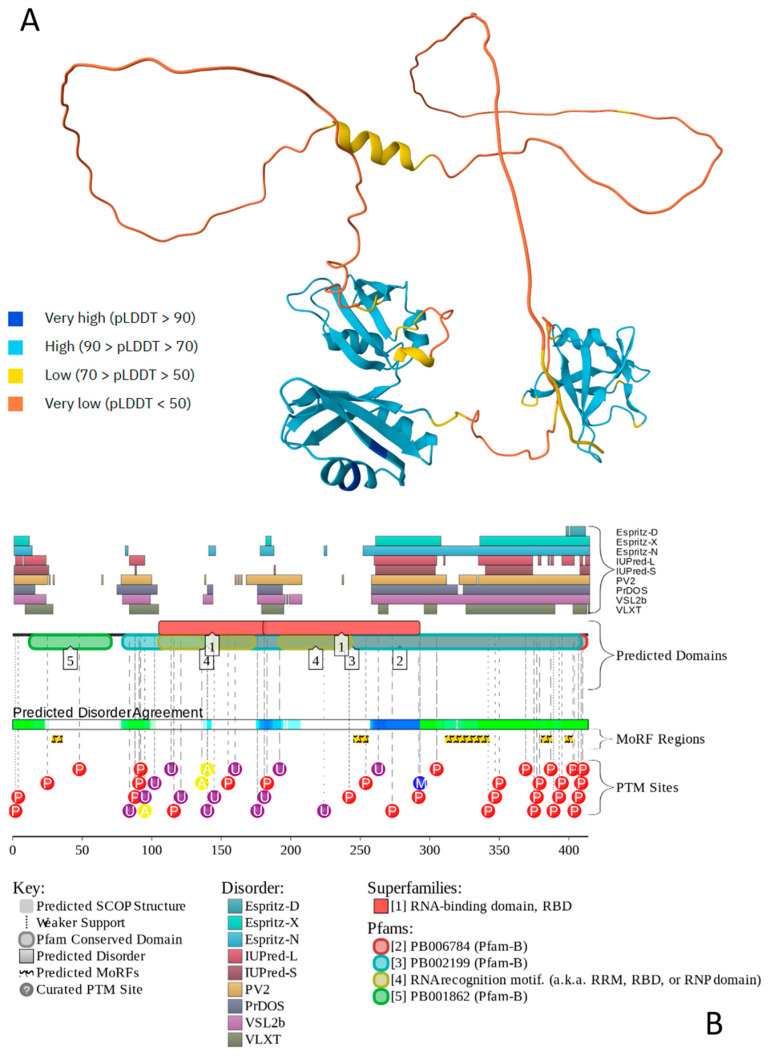
Evaluation of intrinsic disorder in human TAR DNA-binding protein 43 (UniProt ID: Q13148). (**A**) 3D structural model generated for human TDP-43 by AlphaFold. (**B**) Functional disorder profile generated by D^2^P^2^.

Figure 5 illustrates the highly disordered nature of human TDP-43 and shows that its IDRs serve as targets for various PTMs and are also used for disorder-based interaction with partners (as per BioGRID, there are at least 572 protein partners of TDP-43). Furthermore, as per FuzDrop analysis, human TDP-43 has a high probability of spontaneous LLPS (p_LLPS_ = 0.8981) and contains a long, C-terminally located DPR (residues 251–414), confirming that the amino acid sequence features of this protein are consistent with its ability to undergo spontaneous LLPS.

### 2.5. Fused in Sarcoma (FUS)

Fused in sarcoma (FUS), also known as translocated in liposarcoma (TLS), is a nuclear DNA/RNA-binding protein essential for RNA metabolism, the transcription and splicing of mRNA, and stress responses [7,224]. Outside of the nucleus, FUS plays an important role in neurons and is involved in the dendritic maturation and complexity of mouse hippocampal neurons via transporting mRNA to the dendrites [225]. FUS is characterized by an intrinsically disordered structure, which allows it to exist in a dynamic ensemble of conformations rather than adopting a stable 3D structure. The protein has an N-terminal QGSY-rich region, a globular RNA-recognition motif (RRM) with the classical βαββαβ fold, a zinc finger domain (ZnF), three different RGG boxes (RGG 1, RGG 2 and RGG 3), a G-rich region and a C-terminal nuclear localization signal (NLS). The RGG boxes promote the affinity of folded domains for RNA, possibly without taking a defined conformation during nucleotide binding [78,199,226].

The high proportion of Arg-Gly-Gly repeats in the RGG motifs seems to be a key factor for RNA–protein interactions and is predicted to be completely disordered with a high degree of flexibility, which is typical of IDPs. The presence of disordered and low-complexity domains allows FUS to engage in various interactions while retaining flexibility, which is essential for its multifunctional role within the cell during RNA binding [226].

Missense mutations (R521C) in FUS can lead to neurodegenerative diseases such as familial ALS [227,228,229]. Around 5% of mutations in FUS/TLS account for familial ALS [229]. Additionally, mutations in the FUS gene can lead to forms of FTD and FTLD, linking specific sequence variations to pathogenic behavior and further suggesting that the functional capacity of FUS is heavily dictated by its disordered nature [78,230,231]. Sporadic ALS and FTLD often arise from spontaneous mutations. Mutations in FUS/TLS also trigger the degeneration of motor neurons [78,131,227,228]. FUS was also found in neuronal inclusions and shown in previously unrecognized glial pathology. Immunoblot analysis of proteins extracted from post-mortem FTLD patient brain tissue demonstrated increased levels of insoluble FUS, but mutations in the FUS gene were not found [232].

FUS has been demonstrated to undergo LLPS in vitro, and its RNA-binding capacity is closely related to its ability to phase-separate [233,234]. It can form MLOs, which play critical roles in stress response and RNA processing [235,236,237,238,239,240,241,242,243,244,245,246,247]. The ability of FUS to condense into these structures underscores the significance of its dynamic properties in cellular contexts. The transitions between soluble and aggregated states are influenced by its IDRs, highlighting the pathogenic potential of FUS misfolding [105]. PTMs such as methylation, phosphorylation, acetylation, and ubiquitination play a critical role in the regulation of FUS. Interestingly, the phosphorylation of FUS’s low-complexity domain disrupts phase separation, aggregation, and toxicity [237].

TDP-43 and FUS/TLS have striking structural and functional similarities, implicating alterations in RNA processing as a key event in ALS pathogenesis [227,228]. Abnormal aggregation contributes to dysfunctional protein homeostasis and neurodegeneration, with FUS anomalies exacerbating these conditions [7].

These sequence characteristics of FUS and related RNA-binding proteins provide valuable insights into how they can maintain functional flexibility with their ability to misfold and aggregate under pathological conditions. Furthermore, FUS aggregates are able to form cytoplasmic inclusions, which are associated with neurodegeneration [248].

FUS exemplifies how intrinsic disorder can have both effects: the ability to interact with multiple partners for its normal functions and its aggregation capacity, contributing to neurodegeneration. A detailed understanding of FUS’s intrinsic disorder can lead to potential therapeutic approaches.

**Figure 6:**
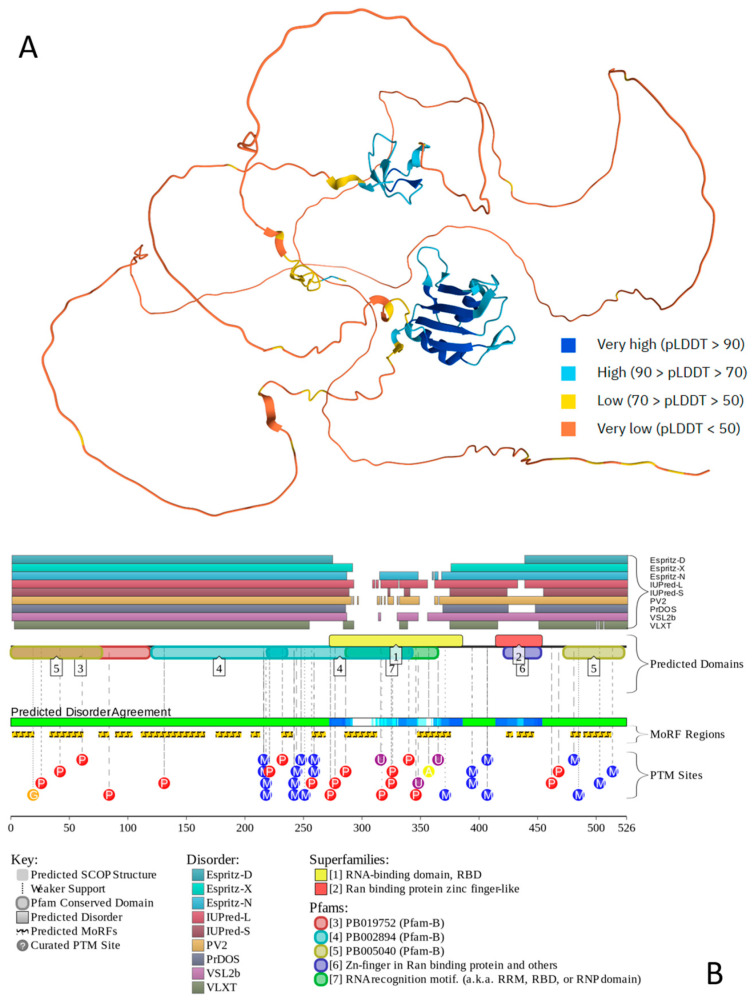
Evaluation of intrinsic disorder in human RNA-binding protein FUS (UniProt ID: P35637). (**A**) 3D structural model generated for human FUS by AlphaFold. (**B**) Functional disorder profile generated by D^2^P^2^.

Figure 6 illustrates the prevalence of intrinsic disorder in human FUS and suggests that structural pliability is important for the function of this protein, which is heavily decorated by multiple different PTMs and contains 15 MoRFs that cover a very significant part of its sequence. Intrinsic disorder can contribute to the multifunctionality of this protein and its ability to interact with multiple partners. According to BioGRID, FUS can interact with almost 850 proteins. FuzDrop analysis revealed that human FUS has an extremely high p_LLPS_ of 0.9999 and contains three DPRs (residues 1–294, 360–437, and 443–526) that cover almost 90% of its sequence, providing strong support to the capability of this protein to act as a powerful droplet driver capable of undergoing spontaneous LLPS.

### 2.6. Commonality of Individualities

Table 1 provides a systematic overview of some of the characteristic features of human Aβ, tau, α-synuclein, TDP-43 and FUS and shows that these proteins exhibit substantial IDRs that support physiological flexibility but are also predisposed to aggregation and cross-interaction under pathological conditions.

### 2.7. Mighty Alliance: Beyond the Individual Armies

**Figure 7:**
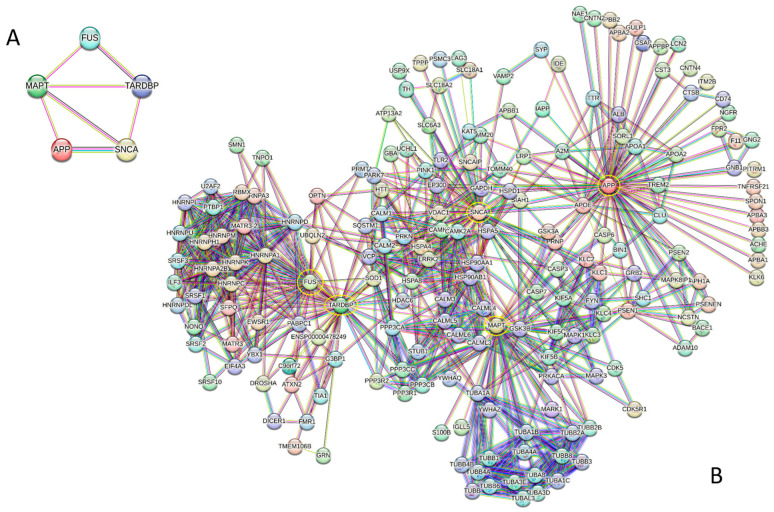
STRING-generated protein–protein interaction (PPI) networks of human proteins linked to mixed pathologies. (**A**) Intraset PPI network connecting APP, tau, α-synuclein, TDP-43, and FUS. (**B**) Joint PPI network centered at five major mixed pathology-related proteins, highlighting shared interactors. Positions of APP, tau, α-synuclein, TDP-43, and FUS within this network are highlighted by yellow circles. This PPI network was generated by the STRING database (https://string-db.org (accessed on 20 January 2026) [252,253,254]) using the maximum number of interactors in the first shell of 500 and a highest confidence level of 0.9 based on a minimum required interaction score. Here, individual proteins act as network nodes, whereas differently colored edges show protein–protein associations based on different types of evidence. The blue line represents information extracted from the curated databases, the black line represents co-expression, and the green line represents gene neighborhoods. Based on the STRING annotations, edges are expected to be specific and meaningful, indicating that linked proteins jointly contribute to a shared function. However, this does not necessarily mean that they are physically binding to each other. The interactive version of the joint PPI network can be viewed at the following permalink: https://version-12-0.string-db.org/cgi/network?networkId=bhz5YRrlPsno (accessed on 20 January 2026).

The facts considered so far illustrate the individual importance of Aβ/APP, tau, α-synuclein, TDP-43, and FUS in both physiological and pathological processes. However, these proteins are not acting alone but interacting with each other, forming a network with seven edges and an average local clustering coefficient of 0.767. Since the expected number of edges for the random network of this size is 0, this intra-set PPI network has significantly more interactions than expected (PPI enrichment *p*-value 3 × 10^−11^) (see Figure 7A).

Crucially, these five proteins not only possess individual “armies” of numerous interactors, but they also exhibit collective interactivity, featuring multiple shared binding partners. This idea is illustrated by Figure 7B, showing that these proteins function not just as individual “hubs” but as a unified group with high interconnectivity. In fact, although this network was generated using high restringing settings (the highest confidence level of 0.9 for a minimal interaction score), it includes 190 proteins involved in 807 interactions, which significantly exceeds the 289 interactions expected to occur in a random set of proteins of the same size and degree distribution drawn from the genome (PPI enrichment *p*-value < 10^−16^). The average local clustering coefficient of this network is 0.654, and its average node degree is 8.5. Among the 190 proteins in this network, 83 have at least eight interacting partners, with 26 proteins interacting with more than 15 partners each. The most significant number of interactors is ascribed to the “army commanders”, APP, MAPT/TAU, SNCA/synuclein, TARDBP/TDP-43, and FUS, which interact with 63, 49, 45, 43, and 34 partners, respectively. Next in the interactivity ranks within this united network are HNRNPA1, HNRNPC, HNRNPM, HSP90AA1, HNRNPA2B1, HNRNPH1, HNRNPK, and GSK3B interacting with 30, 24, 24, 23, 22, 22, 22, and 21 partners. There are also 26 proteins that interact exclusively with one of the “army commanders”. There are no proteins that would serve as joint interactors for five and four connected human proteins linked to mixed pathologies, and only one “outside” protein, APOE, connects three “army commanders” (APP, SNCA, and MAPT). However, TARD and FUS have 15 joint interactors: DROSHA, EWSR1, HNRNPA1, HNRNPA2B1, HNRNPA3, HNRNPC, HNRNPH1, HNRNPK, HNRNPM, MATR3-2, OPTN, RBMX, SFPQ, SOD1, and UBQLN2. IAPP, KLC1, KLC2, and PRNP are common binding partners of APP and SNCA; MAPT and APP share CASP3, GSK3A, and PSEN1; LRRK2, PRKN and VDAC1 are shared by MAPT and SNCA; whereas HSPA4 and HTT serve as joint interactors for TARDBP and SNCA. Obviously, if less restrictive settings are used, the resulting PPI network will include more shared partners. However, conducting such an analysis is outside the scope of this review.

Table 2 illustrates the interactivity of APP, tau, α-synuclein, TDP-43, and FUS and the impressively broad functionality of these proteins and their interactors. Although the data assembled in this table represent a set of “dry numbers”, they reflect an extremely comprehensive picture, where the members of the individual interactomes of these five human proteins linked to mixed pathology and their joint interactome are associated with thousands of biological processes, hundreds of molecular functions, and hundreds of pathological processes. In other words, these proteins are not limited to some specialized roles. Instead, their interactomes overlap significantly, allowing them to influence and to touch a very wide spectrum of cellular processes, thereby providing an important illustration of the “mighty alliance” notion. Therefore, by working together through these dense networks, these proteins create a “comprehensive picture” of highly connected cellular activity, which explains why they are so often linked to mixed pathology in the human brain. In fact, the misfolding of even one key protein can overwhelm cellular proteostasis networks (chaperone and degradation pathways), inducing a collapse that disrupts the folding landscape of other aggregation-prone proteomes and thereby triggering a secondary wave of aggregation among vulnerable proteins [255]. Furthermore, filamentous aggregates of one protein can directly trigger the misfolding and aggregation of other amyloidogenic proteins [256,257,258,259], acting as a template for cross-seeding due to shared structural similarities in the amyloid fibrils [260,261].

It is known that in neurodegenerative diseases, the pathological IDPs (such as Aβ, tau, α-synuclein, TDP-43, and FUS) are “networked”, forming a complex, interconnected web of misfolding, cross-seeding, and co-aggregation. This interactivity is illustrated by several well-known examples:

- Aβ and tau can form soluble complexes promoting their self-aggregation into the insoluble forms observed in AD [262];
- Aβ can trigger tau aggregation [263,264,265,266,267,268,269];
- Tau catalyzes Aβ aggregation and toxicity [270] and acts as the central player of multiple signaling loops in the entangled Aβ–tau cascade [271];
- The synergistic toxicity of Aβ and tau drives mitochondrial dysfunction and neurodegeneration in AD patients while impairing neural circuits in mouse models [130,268,272];
- Aβ, tau, and α-synuclein may interact synergistically to promote each other’s aggregation and accumulation and accelerate cognitive dysfunction [273,274];
- This interplay among Aβ, tau, and α-synuclein is linked to a spectrum of neurodegenerative diseases (Aβ, tau and α-synuclein proteinopathies), including AD, CBD, dementia with DLB, PSP, PiD, and PD, a phenomenon described as triumvirate proteinopathies [274];
- α-Synuclein can be cross-seeded with both Aβ and tau [273,275];
- α-Synuclein and tau are involved in the mechanistic interplay linked to neurodegenerative phenotypes [276], their fibrillar aggregates co-occur in different diseases [277,278], and soluble oligomers of these proteins co-occur in synucleinopathies [279];
- α-Synuclein and tau are involved in direct interactions and form heteroaggregates that exacerbate neurodegeneration [280,281,282,283,284];
- Aβ and α-synuclein can form hybrid oligomers [285,286,287], and α-synuclein can assist in the oligomerization of Aβ [288];
- TDP-43 and FUS tend to co-aggregate or influence each other’s aggregation [201,289];
- TDP-43 is incorporated into FUS assemblies to create rich subcompartments, but FUS does not show the same recruitment into TDP-43 assemblies [290];
- TDP-43 co-aggregates with other pathogenic proteins (Aβ, tau and α-synuclein) and causes co-pathologies in various neurodegenerative diseases (reviewed in [291]);
- Aβ, tau, α-synuclein, TDP-43, and FUS can undergo co-condensation, forming mixed liquid–liquid phase-separated droplets that act as precursors for pathologic amyloid aggregation and “mixed pathology” in patients [202,292,293,294,295,296,297,298];
- At the proteome level, oligomeric Aβ showed widespread effects on TDP-43 in human induced pluripotent stem cells [299];
- The presence of Aβ plaques and tau pathology can directly promote the accumulation of cytoplasmic TDP-43 [300];
- Tau and TDP-43 exhibit pathological synergy, forming shared pathological cascades [195,301];
- TDP-43 can act as a seed, worsening tau pathology and accelerating neurodegeneration [302,303].

As a result of this connectivity, the dysfunction of one protein often impacts others, creating a cascade that spreads through the highly connected, “dense” neural infrastructure of the brain. Recent large-scale proteomic studies and systems biology approaches confirm that proteins function within dense, interconnected networks; analyzing the breakdown of these systems provides a holistic map of neurodegenerative pathology [304,305,306,307,308,309,310,311].

**Figure 8:**
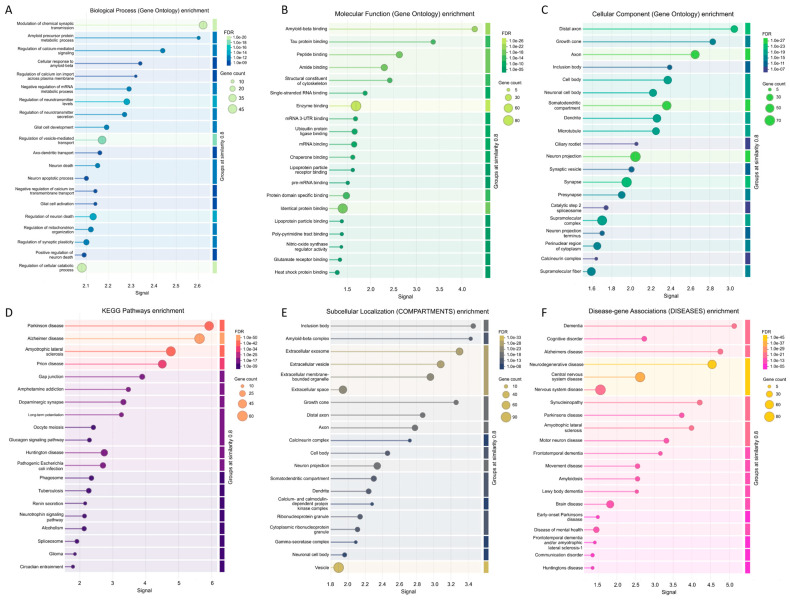
Functional enrichment analysis of the joint interactome of APP, tau, α-synuclein, TDP-43, and FUS. (**A**) Twenty most enriched biological process (BP) Gene Ontology (GO) terms (of the 1055 significantly enriched BP terms). (**B**) Twenty most enriched molecular function (MF) GO terms (of 150 significantly enriched MF terms). (**C**) Twenty most enriched cellular component (CC) GO terms (of 188 significantly enriched CC terms). (**D**) Enrichment analysis of the members of the interactome in KEGG Pathways (20 most enriched of 114 significantly enriched KEGG pathways). (**E**) Enrichment analysis in the subcellular localization (COMPARTMENTS; 20 most enriched of 185 significantly enriched localizations). (**F**) Enrichment analysis of interactome in the disease–gene association (DGA) terms (20 most enriched of 80 significantly enriched DGA terms).

The most enriched biological processes, molecular functions, and cellular components (as per Gene Ontology annotations) of the members of the joined network are shown in Figure 8, along with the most enriched KEGG pathways and the most enriched subcellular localizations and disease–gene associations. Figure 8A shows that among the 1055 significantly enriched biological process GO terms associated with the members of this network, the most enriched are related to the modulation of chemical synaptic transmission, amyloid precursor protein metabolic processes, the regulation of calcium-mediated signaling, cellular responses to Aβ, the regulation of calcium ion import across plasma membrane, the negative regulation of mRNA metabolic process, and the regulation of neurotransmitter levels. Figure 8B shows that among the 150 significantly enriched molecular function GO terms, the most enriched are Aβ binding, tau protein binding, peptide binding, structural constituent of cytoskeleton, and single-stranded RNA binding. As per Figure 8C, of the 188 cellular component GO terms, the most enriched are distal axon, growth cone, inclusion body, cell body, somatodendritic compartment, dendrite, and microtubule. Analysis of the most significantly enriched KEGG pathways revealed that the members of the studied network are related to PD, AD, ALS, HD, prion disease, gap junction, amphetamine addiction, dopaminergic synapse, and long-term potentiation (Figure 8D). Figure 8E shows that among the 186 significantly enriched subcellular compartments are the inclusion body, Aβ complex, extracellular membrane-bounded organelle, growth cone, distal axon, calcineurin complex, and cell body. The multifunctionality of the five analyzed proteins and their interactors indicates that the misbehavior and deregulation of these proteins can be associated with various pathological processes. In agreement with this notion, Figure 8F shows that among the 80 significantly enriched diseases linked to the members of this network are dementia, cognitive disorder, AD, ALS, FTD, PD, neurodegenerative disease, central nervous system disease, motor neuron disease, and synucleinopathy.

**Figure 9:**
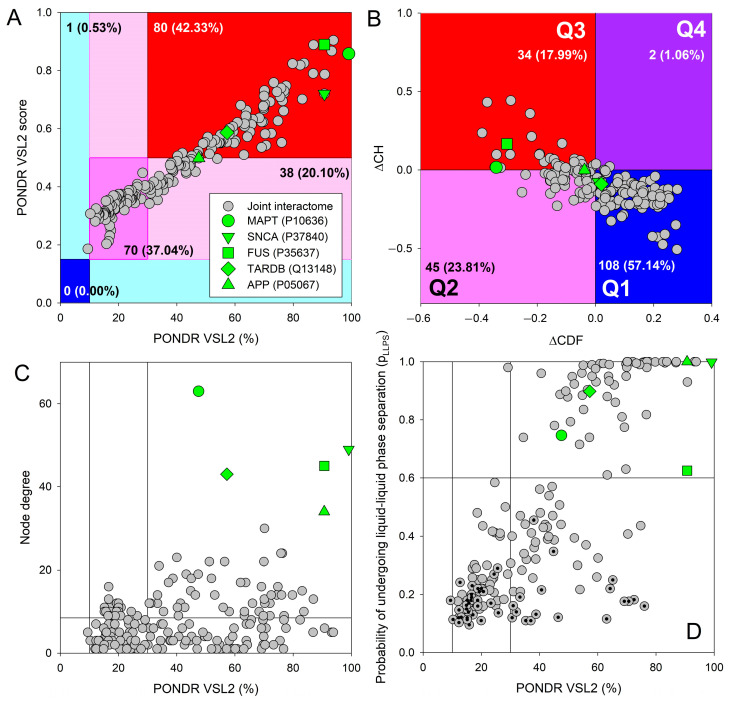
Evaluation of global intrinsic disorder predisposition of human proteins associated with mixed pathologies and their interactors. (**A**) The PONDR^®^ VSL2 score (average disorder score, ADS) vs. PONDR^®^ VSL2 (%) (percentage of predicted intrinsically disordered residues, PPIDR) plot, where each point corresponds to a query protein. In this plot, the coordinates are derived from the PONDR^®^ VSL2 data as the corresponding ADS and PPIDR values. The areas containing highly disordered, moderately disordered, and ordered proteins (based on the accepted classification, see text) are shown by red, pink/light pink, and blue/light blue colors, respectively. The regions where the ADS are PPIDR agree are indicated by dark (blue or pink) colors, whereas light blue or pink colors correspond to the areas where only one of these criteria applies. (**B**) Classification of query proteins based on the outputs of the charge-hydropathy (CH, which correlates a protein’s net charge with its hydrophobicity) and cumulative distribution function analyses (CDF, which correlates the cumulative frequency of disorder scores with the disorder scores). The resulting ΔCH-ΔCDF plot integrates the corresponding outputs as a two-dimensional graph, where the deviation of the disorder frequency of a query protein from the CDF boundary (ΔCDF) is shown on the X-axis, and the distance of a protein from the CH boundary (ΔCH) is shown on the Y-axis. Here, proteins that are expected to be structured, molten globular/hybrid, highly disordered, or mixed are located within Quadrant 1 (blue, **bottom right**), Quadrant 2 (pink, **bottom left**), Quadrant 3 (red, **top left**), and Quadrant 4 (violet, **top right**). (**C**) Correlation between the interactivity (measured as the node degree in the STRING-generated PPI network shown in Figure 7B) and intrinsic disorder (measured as PONDR^®^ VSL2-based PPIDR) among the members of the analyzed interactome. (**D**) Comparison of the LLPS predisposition of the analyzed proteins with their intrinsic disorder propensity. Vertical lines show 10% and 30% PPIDR thresholds, whereas the horizontal line corresponds to the p_LLPS_ threshold of 0.6. Proteins not related to LLPS (showing p_LLPS_ below 0.6 and not containing DPRs) are shown by dotted circles. In these plots, the positions of individual major proteins associated with mixed pathologies are shown by differently shaped green symbols, whereas the gray circles indicate the corresponding data for their interactome.

Importantly, not only APP, tau, α-synuclein, TDP-43, and FUS are characterized by high intrinsic disorder content; many of their interactors are disordered as well. This is illustrated by Figure 9A, representing the PONDR^®^ VSL2 score (average disorder score, ADS) vs. PONDR^®^ VSL2 (%) (percentage of predicted intrinsically disordered residues, PPIDR) plot. Both PPIDR and ADS values are used to rank proteins. Based on their PPIDR scores, proteins are classified as highly ordered, moderately disordered, or highly disordered if their PPIDR values are below 10%, between 10% and 30%, and above 30%, respectively [312,313]. Alternative classification of proteins as highly ordered, moderately disordered/flexible, or highly disordered is derived from their ADS values, with ADS < 0.15, 0.15 ≤ ADS < 0.5, and ADS ≥ 0.5, respectively.

Based on these criteria, almost all interactors are clearly classified as moderately or highly disordered by PONDR^®^ VSL2, with just one protein being predicted as highly ordered by PPIDR and not by ADS (i.e., located within the cyan area). For comparison, the analogous analysis of the entire human proteome (20,317 proteins) revealed that 0.4%, 5.1%, 33.7%, 21.0%, and 39.8% proteins were located within dark blue, cyan, dark pink, light pink, and red areas, respectively [314]. Therefore, with their 0.0%–0.53%–37.04%–20.10%–42.33% distribution, interactors are generally more disordered than the whole proteome. Note that APP, tau, α-synuclein, TDP-43, and FUS are all predicted as highly disordered (see Figure 9). Figure 9B represents the results of global disorder analysis of human proteins interacting with APP, tau, α-synuclein, TDP-43, and FUS in the form of the ΔCH-ΔCDF plot that can be used for further classification of proteins as mostly ordered, molten globule-like or hybrid, or highly disordered based on their positions within the resulting CH-CDF phase space [315,316,317,318]. This analysis provides further support to the idea that the human proteins interacting with APP, tau, α-synuclein, TDP-43, and FUS include noticeable levels of disorder, being a bit more disordered than the human proteome in general, which contains 59.1%, 25.5%, 12.3%, and 3.1% proteins in quadrants Q1, Q2, Q3, and Q4, respectively [314]. Figure 9C shows the correlation between the interactability of the analyzed proteins (in terms of their node degree) and their intrinsic disorder status and demonstrates that there is a weak positive correlation between these two parameters. This is an expected behavior, as disordered proteins are typically prone to being more promiscuous binders. Finally, Figure 9D shows the correlation between the propensity of all the query proteins to undergo LLPS and their intrinsic disorder status in the form of the p_LLPS_ vs. PPIDR plot. This analysis revealed that many studied proteins, which are predicted to be highly disordered, are capable of spontaneous LLPS. In fact, in addition to APP, tau, α-synuclein, TDP-43, and FUS, 60 of their interactors are expected to serve as droplet drivers, and 74 interactors can operate as droplet clients. In other words, only about one-third of these proteins are not related to LLPS.

## 3. And Intrinsic Disorder to Rule Them All

Despite their diverse primary sequences and physiological roles, Aβ, tau, α-synuclein, TDP-43, and FUS share a pronounced degree of intrinsic disorder, low sequence complexity, and enrichment in charged and polar residues. These common biophysical features promote multivalent and dynamic PPIs, extensive PTMs, and participation in highly connected interaction networks. Importantly, the same disorder-driven properties that enable functional flexibility under physiological conditions also predispose these proteins to undergo LLPS and aberrant phase transitions. Consequently, intrinsic disorder provides a mechanistic bridge linking protein-specific biology with the formation of biomolecular condensates and, under conditions of stress or aging, their conversion into pathological aggregates.

Due to structural and functional similarities of IDPs, mixed proteinopathies and neuropathologies occur frequently rather than being rare exceptions [5]. These proteins often share IDRs with amino acid compositions known to promote disorder and can interact through multiple pathways, facilitating their co-aggregation with metabolites shown to form amyloid-like structures in inborn error of metabolism disorders and the potential to promote protein aggregation [319]. Multiple protein aggregations lead to specific symptoms of neurodegenerative diseases. This phenomenon is well studied for the major proteins involved in neurodegeneration, including Aβ and tau in AD, α-synuclein in PD, and TDP-43 in ALS. These proteins are described as intrinsically disordered and possess enhanced ability to partition into biomolecular condensates [320]. This pathological interplay is shown for Aβ, tau and α-synuclein, highlighting the need for a holistic understanding of neurodegeneration [320], which would enhance the understanding of neurodegenerative diseases. The sections below provide more insight into the involvement of intrinsic disorder in various aspects of these proteins. Here, we propose a selectivity model to elucidate the mechanisms underlying mixed proteinopathies in neurodegeneration, structured around three main pillars:

(1) Commonalities in Pathogenic Proteins: Identifying shared structural and functional features among proteins responsible for neurodegeneration;
(2) Mechanisms of Aggregation and Spreading: Highlighting how intrinsic disorder drives co-localization, LLPS, amyloid aggregation, cross-seeding, and prion-like propagation;
(3) Proteostasis and Aging: Analyzing the limitations of cellular proteostasis systems and the impact of aging on the handling of these protein aggregates.

### 3.1. Shared Disorder Features Across Neurodegeneration-Associated Proteins

IDPs in general differ from ordered proteins in their content of charged residues, global amino acid composition, strategic positioning of aromatic residues, and the presence of repeating motifs. All these features define their interaction compatibility, even if there is no universal rule that predicts co-aggregation and mixed pathology. Shared disorder features drive mixed pathology in neurodegeneration. The co-occurrence of neurodegenerative non-AD-type proteinopathies is increasingly recognized to be a frequent event in the brains of symptomatic and asymptomatic patients, particularly in older people [109]. These features are, for example, high conformational flexibility, a lack of entirely or partially stable secondary and 3D structure, and the ability for aggregation. PID may explain the effects of neurodegeneration through shared molecular mechanisms, underscoring their connection. The aforementioned proteins illustrate that high levels of intrinsic disorder and the presence of low-complexity regions are recurrent features, but they occur in distinct forms. This raises the question of which features are actually important for driving the mixed pathology. Definitively, PID is a permissive feature but not the only sufficient explanation for mixed pathology. APP, α-synuclein, and tau are highly disordered, while TDP-43 and FUS are better described as proteins containing IDRs and LCDs [88,161,167,321,322].

### 3.2. Co-Localization Driving Mixed Pathology

The co-occurrence of multiple IDPs within one cell can give valuable insights into mixed pathology. This requires spatial and temporal co-localization of aggregation-prone proteins, which dramatically increases the likelihood of heterotypic interactions and cross-seeding. It was shown that Aβ and tau co-localize, with Aβ pathology often accelerating tau pathology [264]. α-Synuclein can induce the fibrillation of tau, and the combined presence of both proteins synergistically promotes their mutual aggregation [280]. TDP-43 undergoes active axonal transport and co-localizes with other RNA-binding proteins like FUS within cells [196]. Other examples of cooperation between Aβ, tau, α-synuclein, TDP-43, and FUS are provided in Section 2.7. It seems that LLPS plays a key role by concentrating these proteins into specific compartments, thereby increasing the probability of interaction. However, this local compartmentalization does not inherently guarantee direct binding or cross-seeding. Therefore, despite these insights, current research lacks ideal real-world models, making it difficult to firmly establish direct causality in these pathogenic mechanisms.

### 3.3. Intrinsic Disorder and LLPS

Proteins, for example, APP [139], tau [86,145,146,147,148,149,150,151,152,153,154,155,156,157,158], α-synuclein [180,181,182,183,184], TDP-43 [78,103,203,204,205,206,207,208,209,210,211,212,213,214,215,216,224], and FUS [235,236,237,238,239,240,241,242,243,244,245,246,247,323], have been reported to undergo LLPS largely mediated by their IDRs or LCDs, leading to the formation of MLOs that are essential for various biological and cellular functions. IDRs have a possibility to be engaged in weak and multivalent interactions, which enable reversible assembly and dynamic compartmentalization even without membranes in the form of liquid droplets. Stress can drive these normally reversible condensates toward pathology [74,75], and the aberrant forms of condensates are commonly associated with many human diseases, including cancer, neurodegeneration, and infectious diseases [75,320]. LLPS is not a universal, unifying property of all intracellular proteins but rather a specialized mechanism driven by specific sequences and regulatory factors. While many proteins, particularly those containing IDRs, can undergo LLPS to form MLOs or biomolecular condensates, this behavior is highly sensitive to the sequence architecture of a protein (i.e., the presence of LCDs, prion-like domains, RGG motifs, and repetitive charged or aromatic residues), the presence of partners, such as RNA, and cellular stress conditions [88,146]. This process plays an important role in neurodegeneration-associated proteins and their aggregation [320].

### 3.4. Intrinsic Disorder and Aggregation

IDPs are critical for neuronal regulation, yet their lack of a fixed 3D structure makes them prone to misfolding. These proteins can undergo abnormal phase transitions, turning from liquid-like droplets into solid, harmful amyloid aggregates. As a result, many proteins involved in neurodegenerative diseases, including Aβ [119,320,324,325,326], tau [146,327,328,329,330], α-synuclein [144,166,167,186], TDP-43, and FUS [199,238], are highly intrinsically disordered. It is their structural plasticity that enables them to potentially engage in various biological functions, such as cell signaling and transport [331]. For example, the fibrils and oligomers of Aβ_40_, Aβ_42_, and α-synuclein act as seeds, affecting the aggregation pathways of other IDPs [332]. Other evidence shows that α-synuclein can enhance tau inclusions in neurons, indicating that the aggregation of these proteins is not isolated but rather interconnected [190]. This supports the concept of mixed proteinopathies and neuropathologies, wherein multiple proteins can aggregate together, contributing to complex disease manifestations [3,274]. For example, studies with Aβ and tau suggest similar cross-seeding and prion-like propagation mechanisms [333,334]. The propensity of IDPs to aggregate is, however, highly context-dependent, reflecting a delicate balance between remaining soluble and adopting pathogenic aggregated states, which is influenced by environmental factors (pH, temperature), PTMs, and, crucially, interactions with specific partners [323,335]. Importantly, the presence of IDRs alone is not sufficient to explain aggregation, and the mechanisms of co-aggregation remain unclear [335]. Defining a quantitative threshold for aggregation is critical to improving our understanding of this process.

Under pathological conditions, IDPs with their specific IDRs can transition into solid-like states, forming irreversible aggregates that disrupt cellular function. For instance, the aggregation of TDP-43 is largely driven by its C-terminal LCD [336]. In neurodegenerative diseases, this pathogenic transition is concerning. Both tau and FUS proteins tend to form stable, insoluble aggregates that are neurotoxic and impair vital cellular mechanisms [88,103,337]. Understanding how cellular stress triggers pathological phase transitions and breaks down chaperone-mediated stability is crucial for maintaining protein stability in aging neurons and fighting neurodegeneration [86,337,338,339]. In summary, the ability of IDRs to promote cross-seeding and co-aggregation provides valuable insight into how mixed proteinopathies arise during neurodegeneration [332].

### 3.5. Convergence of Aggregation Pathways in Mixed Pathology

The intersection of distinct protein aggregation pathways of different proteins serves as a fundamental driver of neurodegeneration, where misfolded proteins act as seeds, accelerating the aggregation of other, distinct proteins. Shared local structures (motifs) within these proteins function as nucleation sites, acting as a structural “template” that fosters a rapid cross-protein aggregation process that propagates across different protein types [340]. Evidence indicates that major pathological proteins, Aβ, α-synuclein, tau, TDP-43, and FUS, can interact synergistically, directly influencing each other’s aggregation to produce mixed proteinopathies (e.g., AD with Lewy bodies) and more aggressive, accelerated cognitive dysfunction, thereby highlighting the profound interdependency of these molecules in the progression of neurodegenerative diseases [3,333]. The convergence of aggregation pathways, leading to neurodegeneration, is a multifaceted phenomenon and can occur through various mechanisms, including templated seeding, PTMs, and environmental stressors, rather than relying on one solitary pathway (see Section 2.7).

### 3.6. Prion-like Behaviors in Disordered Protein Systems

Recent studies indicate the possibility that mixed pathologies arise from cross-seeding, where aggregated states of one amyloidogenic protein (the “seed”) trigger the fibrillation and aggregation of unrelated proteins in a process characterized by “prion-like” behavior, where the seed is essentially forcing the new monomers to adopt the same fold through a process of templated recruitment. The co-occurrence of misfolded protein aggregates has been described in patients affected by several proteinopathies, suggesting a possible molecular cross-talk between pathological processes associated with different diseases. One putative mechanism for this cross-talk is a direct interaction between misfolded proteins, leading to the cross-seeding of protein aggregation [341]. α-Synuclein demonstrated the cross-seeding of prion proteins, thereby illustrating direct cross-seeding between unrelated amyloidogenic proteins associated with different neurodegenerative diseases [342]. It was also shown that Aβ and α-synuclein aggregates induce the fibrillation of tau, framing neurodegeneration as a collapse of proteostatic networks rather than isolated proteinopathies, showing that aberrant conformations can be transmitted [3,333]. IDPs with their shared structural features aid this process. However, although the in vitro evidence is strong, the in vivo relevance and specific mechanisms governing the “cross-seeding barrier” (i.e., why only some amyloid proteins interact) are not fully understood. In fact, amyloid cross-seeding is not a universal phenomenon. Instead, it is highly selective and seems to be limited by strict structural compatibility, meaning only specific amyloid pairs can seed one another. This selectivity demonstrates the existence of a kind of “cross-seeding barrier,” where successful interaction relies on precise molecular matching, such as the sharing of specific epitope regions, conformational similarity, and complementary electrostatic or hydrophobic interactions. Furthermore, the specific structural arrangements of the starting seeds, or “strains”, act as a precise blueprint that governs both the kinetic efficiency and the final architecture of the resulting amyloid fibers. This self-propagating “seeding effect” allows for the transmission of distinct conformations, with variations in strain structure correlating to different levels of toxicity, mobility, and clinical phenotypes within biological systems. Further research is needed to determine the exact structural basis of these interactions and to develop therapeutic strategies that can target multiple aggregated proteins simultaneously.

### 3.7. Intrinsic Disorder, Aging, and Proteostasis Failure

The connection between intrinsic disorder, aging, and proteostasis failure is central to the development of proteinopathies and neurodegeneration. As cells age, the proteostasis network, a complex cellular mechanism responsible for protein folding, quality control, and degradation, becomes less efficient, leading to the accumulation of misfolded, damaged, and potentially pathogenic proteins. IDPs are particularly vulnerable to these age-related stressors, leading to the stabilization of aberrant condensates that form the toxic aggregates characteristic of diseases and cause the systems-level failures in long-lived neurons [5].

Proteostasis (protein homeostasis) is the cellular process that maintains the proteome in a functional, balanced state by regulating protein synthesis, folding, trafficking, PTMs, and degradation. As this system declines with age, misfolded and aggregated proteins accumulate, particularly damaging non-dividing cells like neurons. This age-related impairment is characterized by a reduced ability of molecular chaperones and degradation pathways to maintain protein quality control [338,343]. The accumulation of misfolded proteins increases the risk of neurodegenerative diseases, such as AD, PD, ALS, and FTD, to name a few [343,344]. Age-related stress, such as oxidative stress, mitochondrial dysfunction, and declining proteostasis, causes IDPs to misfold and aggregate because their high conformational flexibility, lack of stable structure, and binding promiscuity make them inherently sensitive to changes in their environment [88,345]. As a result of the progressive decline of proteostasis, IDPs, which play numerous important roles in cellular functions but can lead to toxicity when deregulated [343], are often “trapped” in disordered, non-native states, resulting in aberrant LLPS or the formation of insoluble amyloid-like aggregates.

Long-lived, post-mitotic neurons face significant proteostasis challenges because they cannot dilute accumulated protein damage through cell division. As neurons age, mechanisms for protein folding and clearance (e.g., chaperones, autophagy) decline, leading to toxic, misfolded aggregate accumulation that can interfere with neuronal functions [338,346]. Aging-related decline in the proteostasis network, particularly under stress, leads to an inability to manage the accumulation of misfolded proteins and cytotoxic protein aggregates. This failure in handling intrinsic disorder results in neuronal toxicity, a key driver of neurodegenerative disease pathogenesis [338,343,347]. Aging alters the regulation and patterns of PTMs, which affect, control, and regulate the solubility, localization, and aggregation propensity of proteins. Proteostasis failure does not affect all proteins equally, and current research suggests that defined thresholds exist at which the proteostasis network collapses, driving irreversible protein aggregation. While proteostasis decline is a general feature of aging, its impact is highly dependent on protein stability, abundance, and the specific cell type, with long-lived neurons being particularly sensitive [348].

### 3.8. Implications for Disease Classification and Mechanistic Understanding: Rethinking Neurodegenerative Diseases as Intrinsic Disorder-Driven Network Failures

While the aforementioned pathological pathways provide a common mechanistic framework for neurodegeneration, the specific disease entity is dictated by a complex interplay of intrinsic genetic factors and extrinsic environmental influences. Neurodegenerative disorders can be comprehensively recontextualized as the failure of intrinsic disorder-driven networks. Within this framework, specific protein sequences, LLPS, “cross-seeding” between different proteins, and the breakdown of proteostasis act as the primary drivers of decay [345]. This perspective emphasizes the central role of IDPs and their conformational transitions in the initiation and progression of neurodegeneration in general and mixed proteinopathies in particular. Considering these diseases as systems-level failures of intrinsic disorder-mediated processes represents a more holistic approach, as such a “disordered-network” framework shifts the focus from looking at individual protein aggregates, such as amyloid plaques or tau tangles, as isolated culprits to seeing them as symptoms of a broader failure in cellular biophysics. By viewing these diseases through the lens of IDPs, LLPS, and proteostasis, we can better understand why different neurodegenerative conditions often overlap, giving rise to mixed proteinopathies. This suggests that future therapies may need to move beyond “one drug, one target” and instead aim to stabilize the cellular environment or modulate the physical properties of disordered proteins to prevent them from transitioning from functional droplets into toxic solids.

### 3.9. Selectivity Model to Explain Mixed Pathology

All these points can be summarized in a selectivity model that offers a framework for understanding the mechanisms driving mixed proteinopathies in neurodegenerative diseases, proposing that co-pathology arises from the convergence of three key pillars. Here, rather than independent occurrences, mixed pathologies require the alignment of biophysical compatibility (determined by amino acid sequence features, such as given by the “stickers and spacers” model of amino acid sequence that dictates whether different pathogenic proteins can co-condense or cross-seed; with aromatic and arginine residues often acting as stickers that drive heterotypic interactions) [92,323,349], spatial co-localization (facilitated by LLPS that drives formation of different MLOs and provides a mechanism for sequestering multiple aggregation-prone proteins into the same high-density compartment. For example, stress granules, as a form of MLO, frequently act as “staging areas” for misfolded proteins) [7], and a specific proteostasis context (impaired clearance, aging, and cellular stress), as the inability of the cell to remove misfolded proteins due to aging or stress, lowers the threshold for co-aggregation, allowing co-existing pathologies to mature within the same cellular environment [350]. This model hypothesizes that mixed proteinopathies are not coincidental but occur only when all three components align. To validate this model, future research must quantitatively determine the interplay and relative contribution of these factors in vivo using comprehensive, high-resolution imaging and multi-component model systems.

## 4. Potential Therapeutic Implementations of Intrinsic Disorder

### 4.1. Challenges of Targeting IDPs

Although IDPs hold great potential for treating cancer, neurodegeneration, viral infections, and many other maladies, their lack of a stable 3D structure makes them difficult to target using a traditional structure-based “lock-and-key” drug design. Indeed, how do you fit a key into a lock that is constantly changing its tumblers? Some of the major challenges associated with designing a drug for a “protein cloud” that constantly changes shape are briefly outlined below.

IDPs lack a stable 3D structure under physiological conditions, being characterized by high structural complexity and existing as dynamic conformational ensembles at the edge of chaos [28]. Conventional drugs target deep, hydrophobic pockets, such as active sites of enzymes, allowing the highly specific, high-affinity binding (nM range) required for drugs to be effective at low doses with reduced off-target side effects. However, because of their highly flexible nature, IDPs/IDRs lack those stable, deep binding sites, typically possessing only shallow, solvent-exposed surfaces instead. This lack of a permanent, deep pocket makes it difficult for small molecules to get the necessary surface area contact to achieve high-affinity binding, resulting in weak, micromolar (μM) to millimolar (mM) affinities [351,352,353,354,355,356].

The conformational flexibility allows IDPs to act as highly promiscuous binders, interacting with a broader range of partners than typical ordered proteins [11,49,56,141,142,143] and efficiently orchestrating the activities of their numerous partners [357,358,359,360]. By acting as hubs in signaling protein–protein interaction networks, IDPs interact with many different partners [361]. Targeting a hub protein to block one interaction often accidentally disrupts other vital, normal functions, leading to poor specificity and high side effects [362].

IDPs show a unique ability to undergo conformational changes in different environmental conditions. This is known for interactions with various partners, including small molecules, nucleic acids, membranes, and other proteins [9,10,11,13,14,15,16,49,52,56,363,364,365,366,367]. The conformation of proteins ranges from loosely organized to tightly compact [14,46,54,368]. Furthermore, many IDPs do not follow the “one structure—one function” paradigm by adopting different conformations when binding to different partners [52,369], thereby directly contradicting the “you cannot kill two birds with one stone” rule, and proving that a single protein can indeed serve multiple roles. This highly adaptive nature of IDPs/IDRs poses a challenge for drug design, since a therapeutic molecule must outcompete the natural partner binding, which is generally faster and stronger than binding to a single, fleeting conformation in the “cloud” [356,370,371].

This functional versatility stems from specific intrinsic features, such as exceptional spatiotemporal heterogeneity, where proteins act as “structural mosaics”. These mosaics consist of diverse functional modules, including ordered foldons, disorder-to-order binding modules (inducible foldons), partner-dependent morphing inducible foldons, and order-to-disorder activation modules (unfoldons). This heterogeneity enables a “structure–function continuum”. Distinct regions of proteins perform specialized roles based on their level of disorder [143,372,373,374]. Various PTMs [17,71,72,73,375,376] and alternative splicing [377,378,379] regulate and control the biological activities of IDPs/IDRs. These are two important factors contributing to the complexity of proteomes, where a single gene is known to encode for multiple proteoforms [380,381]. Obviously, these intrinsic features complicate the druggability of IDPs [61,382,383].

Besides their lack of stable 3D structures, high conformational flexibility, “protein cloud” nature, rapid switching between various conformations, multifunctionality, environmental sensitivity, high binding promiscuity, and lack of “deep” binding pockets, which all render conventional structure-based drug design (e.g., docking) ineffective, IDPs have other features that make them difficult drug targets. For example, IDPs/IDRs are often highly hydrophilic, being generally enriched in charged and polar residues, making hydrophobic interaction-driven binding thermodynamically unfavorable [384,385,386,387]. Furthermore, due to their conformational flexibility, these proteins are highly susceptible to proteolysis, creating additional challenges not only for studying them in vitro but also for their therapeutic stability in vivo, where they can be quickly targeted by cellular proteases [388,389]. Another complication stems from the ability of IDPs to undergo LLPS and form various MLOs or biomolecular condensates. The environment within these liquid droplets (such as water activity, viscosity, pH, ionic strength) is different from the cytoplasm or nucleoplasm. As a result, drugs do not distribute evenly between the cytoplasm and the droplet. Since they partition based on their solubility, charge, and hydrophobicity, their effective concentration might be 100 times higher inside a condensate than in the surrounding cytoplasm/nucleoplasm. In other words, the affinity and concentration of drugs can be altered due to LLPS, making it hard to predict drug efficacy in a cellular environment [98,390,391,392,393,394,395,396].

### 4.2. Disorder-Aware Drug Discovery and Intervention Approaches

Despite all the aforementioned challenges, at least some of the IDPs/IDRs can be targeted. In fact, although IDPs/IDRs lack a fixed structure, during interaction with their specific partners, they often form transient localized structures, such as α-helices or β-strands, which can also exist within the conformational ensembles of IDPs/IDRs as transiently populated pre-folded (or pre-structured) motifs. Such transient localized conformations may act as specific targets for small-molecule stabilization [397,398,399]. Such targeting can utilize several specific mechanisms, such as conformational selection via binding and stabilizing the transient pre-structured motifs, thereby shifting the equilibrium toward the more ordered state, or target sequestration by “trapping” the IDP/IDR in a shape that is either compatible or incompatible with its natural binding partner [353,397,398,399].

Another useful strategy is based on inhibiting PPIs by targeting the folded/ordered partner of an IDP/IDR. Since small molecules can bind to the well-defined, structured binding groove on the ordered partner protein to prevent the binding of specific disordered motifs (molecular recognition features), they can effectively disrupt the PPI without directly targeting the disordered region itself. This approach represents a crucial, high-affinity strategy in drug discovery, where traditional structure-based drug design techniques can be used to identify small molecules targeting such stable binding pockets [108,400,401,402].

Targeting IDPs/IDRs with covalent inhibitors is a powerful strategy for dealing with these “undruggable” targets. By establishing a stable chemical bond with specific residues (typically cysteine (C) is taken as the preferred target due to its high nucleophilicity and low natural abundance, which helps minimize off-target effects), these inhibitors bypass the need for traditional binding pockets, ensuring superior potency and extended target engagement compared to non-covalent ligands [403]. Such covalent inhibitors utilize a two-step mechanism, where the molecule first establishes a weak, reversible non-covalent bond with a specific motif in the IDP/IDR, and subsequently, a reactive “warhead” on the drug anchors to a nearby cysteine, forming a permanent or reversible bond that locks the inhibitor in place and inactivates the protein [403,404].

An alternative approach is given by the utilization of PROTACs (proteolysis targeting chimeras) [405] or other forms of targeted protein degradation (TPD) [406], such as lysosome-targeting chimeras (LYTACs) [407], Trim-Away [408], autophagosome-tethering compound (ATTEC) [409], autophagy-targeting nanobody chimera (ATNC) [410], and proteolysis targeting nanobody conjugate (PROTNC) [411], which represent a groundbreaking “event-driven” therapy that completely removes a target protein rather than just temporarily blocking its function. These approaches are based on the utilization of the ubiquitin–proteasome system (UPS), lysosome-endocytosis, or autophagy pathways to clear target proteins [412,413]. This method is especially powerful for targeting IDPs, which were labeled “undruggable” because they lack the stable structural pockets required for traditional drugs to bind.

Also, targeting the charged or polar surfaces of IDPs/IDRs marks a departure from traditional drug discovery, which typically uses the hydrophobic effect to secure molecules in deep, non-polar pockets. Since IDPs/IDRs lack these stable hydrophobic cores and instead feature abundant polar and charged residues, modern therapeutic strategies are shifting toward hydrogen bonding and electrostatic steering to facilitate binding [414,415,416].

It is clear that in the field of neurodegeneration, the intrinsic disorder-aware drug discovery efforts should target conformational ensembles rather than single states, take into account the reality of the interference with multiple binding partners [417], and look for means to block or interfere with the prion-like domains (PrLDs) to prevent pathology spreading [127] and modulate enzymes catalyzing PTMs, such as acetyltransferases, kinases, or phosphatases [418].

Furthermore, possible therapeutic strategies for diseases linked to protein aggregation should follow two primary tracks: (1) the modulation of phase behavior to prevent the formation of harmful structures (e.g., the stabilization of liquid-phase condensates, regulation of PTMs, or inhibition or beneficial alteration of LLPS) [103,336,419,420,421,422,423,424,425] and (2) the enhancement of proteostasis (e.g., enhancing chaperon activity, promoting disaggregation machinery, or activating ubiquitin-proteasome pathways to clear aggregated proteins) [338,343,422,423,426,427]. These and related strategies may help reduce the harmful effects of protein aggregates in neurodegeneration.

### 4.3. Integrating Intrinsic Disorder into Models of Protein-Specific Diseases

Current models of disease progression often focus on specific proteins, which may overlook the contributions of intrinsic disorder and their interconnection. To overcome this, the field should adopt a system-level approach that uses intrinsic disorder as a central framework. This shift from a single-protein-centric model to a system-level framework centered on intrinsic disorder offers a more holistic understanding of how diseases, particularly neurodegeneration, progress. Since IDPs and IDRs are fundamental regulators in signaling and act as “hubs” in interaction networks, adopting this perspective provides several critical advantages.

One of those advantages is improved understanding of the complex, multi-step pathogenesis of neurodegenerative diseases [428]. Unlike ordered proteins and domains, IDPs and IDRs can adopt different structures depending on their environment and binding partner, so a system-level view helps track these conformational changes, revealing how a single protein can trigger different, or even contradictory, downstream pathways during different stages of disease [166]. Furthermore, a systemic approach illuminates the transition from “functional” disordered monomer to “pathological” amyloid fibril, highlighting the environmental factors (pH, crowding, chaperones) that drive this shift [17,429,430].

A holistic understanding of PID offers the benefit of the identification of network reconfiguration by highlighting system hubs as points of extreme vulnerability. In this context, mutations or PTMs can restructure PPIs. Alternative splicing of proteins often targets IDRs, and system approaches can track those proteins [28,431,432,433,434].

System approaches can also enhance drug discovery and therapeutic possibilities. IDPs/IDRs often form “fuzzy” complexes, maintaining their flexibility even after binding. By taking this fuzziness into account and by targeting these fluid, dynamic interfaces rather than traditional static pockets, one can design small molecules that more effectively disrupt PPIs [40,46,435,436,437,438]. A systemic view shows how cellular chaperones normally protect IDPs. Disease is often a failure of this system, and targeting chaperones or the “nanny” proteins that protect IDPs is a better strategy than trying to correct a single misfolded protein [439].

At least in part, IDPs/IDRs function as master regulatory hubs due to their high density of different PTM sites, such as phosphorylation and acetylation, to name a couple. By acting as molecular “rheostats” or “switches,” these modifications tune the charge, shape, and binding preferences of a protein, thereby representing shifts that can fundamentally drive or disrupt disease progression [71,72,73]. Because PTMs are dynamic and often exist as complex combinations, only the system-level, multi-PTM proteomic approaches allow for the detection and interpretation of these important functional shifts and provide means to decode the vital PTM code, determining how these flexible regions integrate multiple cellular signals into a single functional output [440,441,442].

As was already emphasized, many disease-related IDPs, such as those involved in mixed pathology (i.e., tau, α-synuclein, FUS, or TDP-43), are central to the formation of various liquid droplets, e.g., stress granules. From a systems perspective, pathology often stems from LLPS deregulation [443,444]. This can trigger a transition toward “solidification” or pathological aggregation, which represents a structural shift that remains largely invisible to traditional structural biology techniques and requires a system-level approach that involves analyzing, modeling, and targeting the aberrant formation of biomolecular condensates across the molecular, cellular, and organismal levels [303,445].

Finally, a system-level, disorder-based approach allows capturing how the unique nature of IDPs/IDRs turns their greatest asset, structural flexibility, into a primary driver of pathology. This is done by integrating dynamic factors that are often overlooked, such as aging and stress, where age-related decline in proteostasis promotes the accumulation of damaged disordered proteins; cumulative protein burden, where the accumulation of misfolded IDPs overloads cellular degradation machinery, leading to a cascade of proteostasis failure; and molecular mimicry in IDPs, which facilitates the hijacking of regulatory networks in neurodegeneration by exploiting the structural flexibility and functional promiscuity of these proteins to replace physiological regulators and subvert the cellular processes [338].

In summary, viewing diseases through the lens of intrinsic disorder reveals them not as localized malfunctions of a single protein but as the collapse of a flexible, coordinated, and highly interconnected interactive “social network” of the cell.

### 4.4. Biomarkers in Neurodegeneration Considering Intrinsic Disorder

Intrinsic disorder can explain neurodegeneration, and research about it is transforming biomarker identification by shifting focus from rigid protein structures to dynamic conformational ensembles [446]. The high sensitivity of IDPs to environmental factors can act as potential indicators for early pathological changes. These proteins are significantly accumulated in the blood and cerebrospinal fluid (CSF) of patients with neurodegenerative diseases. For example, nearly 75% of proteins in Aβ-specific aggregates are elevated in the serum of Alzheimer’s patients, highlighting their potential as a source of non-invasive biomarkers [446].

IDPs/IDRs are highly susceptible to various modifications, such as excessive PTM. Because these modifications occur in response to cellular stress or pathological environments, they act as early, sensitive molecular biomarkers [447,448,449]. Therefore, PTMs show the “canary in the coal mine” effect, since targeting these specific modifications (using specialized antibodies or mass spectrometry) potentially allows for the detection of “pre-symptomatic” disease states [450,451].

## 5. Open Questions and Future Directions

Despite strong associations and correlations between PID and neurodegeneration, major conceptual gaps remain. For tau, α-synuclein, TDP-43, and FUS, it is unresolved which disordered conformational sub-ensembles are toxic and how physiological phase separation transitions into irreversible aggregation. Furthermore, it is still unclear whether intrinsic disorder itself enables cross-seeding and mixed pathology or merely amplifies vulnerability under conditions of aging and proteostasis decline. A further unresolved issue is why broadly expressed disordered proteins produce highly selective neuronal degeneration. Aβ, while less intrinsically disordered, may act as an initiator that destabilizes cellular environments rich in disordered proteins. Overall, intrinsic disorder appears to function as a risk amplifier rather than a singular cause, with pathogenic outcomes emerging from context-dependent failures of regulation, buffering, and cellular resilience.

### Quantitative Thresholds Between Functional Disorder and Pathology

A central unresolved question is whether a quantitative or qualitative threshold of intrinsic disorder exists that permits prediction of neuropathological severity or clinical disease. Importantly, the presence of neuropathological proteins in the brain does not uniformly translate into disease, and conversely, clinical neurodegenerative syndromes do not always correspond to a single, well-defined pathology. Much of the current prevalence data relies on post-mortem studies with limited sample sizes and substantial uncertainty arising from retrospective assessment, comorbidities, and confounders. These limitations complicate efforts to draw causal or predictive conclusions and highlight the need for longitudinal and ethically conducted human studies incorporating a risk–benefit assessment with risky elements.

Another major open issue concerns the determinants of reversibility versus irreversibility in protein condensates. Experimental and pathological studies suggest that IDPs can form dynamic, reversible condensates under physiological conditions but may transition into irreversible aggregates in disease. Factors implicated in this transition include: (1) biophysical properties such as composition, charge distribution, pH, and ionic environment; (2) cross-seeding and direct interactions between different disordered proteins that co-localize within the same cellular compartments; (3) pathological synergy arising from the simultaneous presence of multiple proteinopathies; (4) shared upstream stressors, including inflammation, oxidative stress, and proteostasis failure; and (5) shared downstream consequences such as synaptic dysfunction and neuronal loss. The relative contribution of these factors, and their interaction over time, remains incompletely understood, and this knowledge could be an important advantage for systems neuroscience.

## 6. Conclusions

### 6.1. Intrinsic Disorder as a Unifying Biophysical Principle

This review highlights PID as a unifying biophysical framework for the understanding of neurodegenerative disease. IDPs play essential physiological roles in the brain, where synaptic plasticity, rapid signaling, and dynamic PPI require structural flexibility. However, these same properties render such proteins vulnerable to dysregulation, misfolding, and pathological interaction. The evidence reviewed here suggests that PID does not represent a pathological feature, per se, but rather a context-dependent risk factor whose consequences depend on cellular environment, aging, and network-level interactions.

### 6.2. From Isolated Proteinopathies to Interacting Disorder-Driven Networks

The isolated look at proteinopathies without considering interacting neuropathological proteins and their PID leads to a misunderstanding of the whole picture and must lead to a holistic understanding of neuropathology and neurodegeneration, especially in an aging population.

### 6.3. Outlook for Disorder-Centric Neurodegeneration Research

The understanding of interacting disorder-driven networks can lead to research that could consider determinants of molecular biology, biophysics, and interconnectedness to move basic research forward and convert it into treatment options in an interdisciplinary manner.

## Figures and Tables

**Table 1:** Comparative overview of major neurodegeneration-associated proteins with respect to intrinsic disorder, aggregation behavior, phase separation, and contribution to mixed neuropathology.

Feature	Amyloid-β (Aβ) [115,117,119,249]	τ-Protein (Tau) [28,163]	α-Synuclein [144,190,250]	TDP-43 [219,222,251]	FUS [223,227,228]
Physiological role	APP-derived peptide; modulates synaptic activity at low concentrations	Microtubule-associated protein stabilizing neuronal function	Presynaptic protein regulating vesicle trafficking	RNA-binding protein regulating splicing and RNA metabolism	RNA/DNA-binding protein involved in RNA metabolism and stress responses
Pathological aggregate	Extracellular plaques; soluble toxic oligomers	Intracellular neurofibrillary tangles (NFTs)	Lewy bodies and Lewy neurites; oligomers	Cytoplasmic inclusions with nuclear depletion	Cytoplasmic inclusions due to nuclear clearance defects
Intrinsic disorder features	Intrinsically disordered as a monomer	Highly intrinsically disordered; conformationally flexible	Almost entirely intrinsically disordered	Large intrinsically disordered regions	Highly intrinsically disordered; prion-like domains
Sequence determinants of disorder and disorder type	Charged and flexible N-terminus; hydrophobic C-terminal motifs (Aβ_42_)	Low sequence complexity; polar and charged amino acid residues	NAC region; polar and charged amino acid residues, particularly in its N-terminal region	Low-complexity C-terminal domain with polar and charged amino acid residues; charge asymmetry	Low-complexity, Arg-Gly-Gly repeats in the RGG motif
Post-translational modifications (PTMs)	Phosphorylation and acetylation	Hyperphosphorylation and acetylation	N-terminal acetylation and phosphorylation	Hyperphosphorylation and ubiquitination	PTMs modulate phase behavior and aggregation
Phase separation (LLPS) and spontaneous LLPS potential (p_LLPS_)	No classical LLPS; APP can undergo LLPS, aggregation emerges from disordered peptide plasticity; APP p_LLPS_ = 0.7463	Undergoes LLPS; droplets can mature into fibrillar aggregates; p_LLPS_ = 0.9985	Forms stress-induced condensates; p_LLPS_ = 0.6249	Stress-granule–associated LLPS precedes aggregation; p_LLPS_ = 0.8981	Prominent LLPS; condensate hardening linked to disease; p_LLPS_ = 0.9999
Aggregation drivers	Hydrophobic C-terminal region, increased concentration, pH, ionic conditions	PHF6/PHF6* motifs, hyperphosphorylation, increased concentration	Increased concentrations, membrane interaction, stress	C-terminal low-complexity domain, phosphorylation	Low-complexity domain-mediated interactions, mutations (ALS-associated), RNA homeostasis, stress
Prevalence in aged brain	Very high	Very high	Moderate to high	Common, especially in elderly	Rare in community autopsy series
Common co-pathologies	Tau, α-synuclein, TDP-43, FUS, vascular pathology	Aβ, α-synuclein, TDP-43	Aβ, tau	Aβ, tau, α-synuclein	TDP-43; ALS/FTLD spectrum
Cross-seeding/synergy	Seeds tau misfolding; interacts with α-synuclein	Synergizes with Aβ; interacts with α-synuclein	Cross-seeds tau and Aβ; strain-dependent	Often superimposed on AD pathology	Stress-granule and RNA-metabolism interactions
Impact on mixed-pathology dementia	Early driver and amplifier of downstream proteinopathies	Strong determinant of cognitive decline and neuronal loss	Exacerbates cognitive and neuropsychiatric symptoms	Accelerates dementia severity and memory impairment	Associated with earlier onset and aggressive disease
Associated diseases	AD, cerebral amyloid angiopathy (CAA), Down syndrome, Aβ–related angiitis (ABRA), CAA-related inflammation, cerebral amyloidoma, dementia with Lewy bodies (DLB), retinal disorders, traumatic brain injury (TBI)	AD, progressive supranuclear palsy (PSP), corticobasal degeneration (CBD), Pick’s disease (PiD), frontotemporal dementia with Parkinsonism-17 (FTDP-17), argyrophilic grain disease (AGD), chronic traumatic encephalopathy (CTE), Down syndrome, Guam Parkinsonism–dementia complex, postencephalitic Parkinsonism, DLB, PD	PD, DLB, multiple system atrophy (MSA), pure autonomic failure (PAF), REM sleep behavior disorder (RBD), AD, Gaucher’s disease, neuroaxonal dystrophy, neurodegeneration with brain iron accumulation (NBIA)	ALS, FTLD-TDP, LATE, AD, CTE, LBD, Huntington’s disease (HD), multisystem proteinopathy (MSP), Perry syndrome, Alexander disease	ALS-FUS, FTLD-FUS, neuronal intermediate filament inclusion disease (NIFID), basophilic inclusion body disease (BIBD), essential tremor (ET), polyglutamine (PolyQ) diseases (HD and spinocerebellar ataxias SCA1 and SCA3), cancers (myxoid liposarcoma, Ewing sarcoma, acute myeloid leukemia (AML)

**Table 2:** Functional enrichment analysis of the individual interactomes of five human proteins linked to mixed pathology (APP, tau, α-synuclein, TDP-43, and FUS) and their joint interactome. PPI networks were generated by STRING using the maximum number of interactors in the first shell of 500 and a highest confidence level of 0.9 based on a minimum required interaction score.

	APP	Tau	SNCA	TDP-43	FUS	Joint Network
Number of Interactors	64	50	46	44	35	190
Functional Term	Number of statistically significantly enriched functional terms					
Biological Process (Gene Ontology)	476	555	553	243	95	1055
Molecular Function (Gene Ontology)	49	64	65	35	22	150
Cellular Component (Gene Ontology)	89	88	88	52	28	180
Local Network Cluster (STRING)	14	12	9	12	10	35
KEGG Pathways	10	128	53	35	3	174
Reactome Pathways	97	202	26	14	4	295
Disease–Gene Associations (DISEASES)	54	38	48	48	34	80
Tissue Expression (TISSUES)	110	100	85	73	59	178
Subcellular Localization (COMPARTMENTS)	93	92	89	61	39	185
Human Phenotype (Monarch)	76	163	200	243	124	405

## Data Availability

No new data were created or analyzed in this study. Data sharing is not applicable to this article.

## Abbreviations

The following abbreviations are used in this manuscript:

Aβ	Amyloid-β
AD	Alzheimer’s disease
ADS	Average disorder score
AGD	Argyrophilic Grain Disease
ALS	Amyotrophic lateral sclerosis
APP	Amyloid precursor protein
BC	Biomolecular condensate
CAA	Cerebral amyloid angiopathy
CBD	Corticobasal degeneration
CDF	Cumulative Distribution Function
CH	Charge-hydropathy
CSF	Cerebrospinal fluid
CTE	Chronic traumatic encephalopathy
DLB	Dementia with Lewy Bodies
DNA	Deoxyribonucleic acid
FTD	Frontotemporal dementia
FTLD	Frontotemporal lobar degeneration
FTLD-FUS	Frontotemporal lobar degeneration with FUS pathology
FTLD-TDP	Frontotemporal lobar degeneration with TDP-43 pathology
FUS	Fused in sarcoma
IDP	Intrinsically disordered protein
IDR	Intrinsically disordered region
LATE	Limbic-predominant age-related TDP-43 encephalopathy
LBD	Lewy Body Disease
LCD	Low-complexity domain
LLPS	Liquid–liquid phase separation
MoRF	Molecular recognition feature
mRNA	Messenger Ribonucleic acid
MLO	Membrane-less organelles
MSA	Multiple system atrophy
NFT	Neurofibrillary tangle
NAC	Non-Aβ component
NACP	Non-Aβ component precursor
NLS	Nuclear localization signal
PAF	Pure Autonomic Failure
PD	Parkinson’s Disease
PID	Protein intrinsic disorder
PiD	Pick’s disease
PPI	Protein–protein interaction
PPIDR	Percent of predicted intrinsically disordered residues
PrLD	Prion-like domain
PSP	Progressive supranuclear palsy
PTM	Post-translational modification
Q	Quadrant
RBD	Rapid Eye Movement (REM) Sleep Behavior Disorder
REM	Rapid Eye Movement
RNA	Ribonucleic acid
RRM	RNA recognition motif
*SNCA*	Synuclein alpha gene
TAR	Transactive response
*TARDBP*	Transactive response DNA-binding protein gene
Tau	τ-protein
TDP-43	TAR DNA-binding protein 43
TLS	Translocated in liposarcoma
VBI	Vascular Brain Injury
ZnF	Zinc finger domain
3D	Three-dimensional
Amino acids	
A	Alanine
R	Arginine
N	Asparagine
D	Aspartic acid
C	Cysteine
E	Glutamic acid
Q	Glutamine
G	Glycine
H	Histidine
I	Isoleucine
L	Leucine
K	Lysine
M	Methionine
F	Phenylalanine
P	Proline
S	Serine
T	Threonine
W	Tryptophan
Y	Tyrosine
V	Valine
